# Supplementary material for: Dynamically stable radiation pressure propulsion of flexible lightsails for interstellar exploration
Source: Nat Commun. 2024 May 17;15:4203. doi: 10.1038/s41467-024-47476-1 (PMC11101440; doi:10.1038/s41467-024-47476-1)
Supplement: Supplementary file 1 — Supplementary Information [file 41467_2024_47476_MOESM1_ESM.pdf]

Supplementary Information

for the manuscript

Dynamically Stable Radiation Pressure Propulsion of Flexible Lightsails for Interstellar Exploration

Ramon Gao[†], Michael D. Kelzenberg[†], and Harry A. Atwater[*]

Thomas J. Watson Laboratory of Applied Physics
California Institute of Technology
Pasadena CA 91125, USA

[†]These authors contributed equally to this work

*Email: haa@caltech.edu

Table of Contents

Supplementary Note 1: Discussion of Materials' Properties 3
Supplementary Table 1. Mechanical and thermal properties for candidate lightsail materials 4

Supplementary Note 2: Stationary burst diameter figure-of-merit 5
Supplementary Table 2. Mesh simulator burst-intensity results 6
Additional stability considerations 7

Supplementary Note 3: Mesh-based simulator for flexible lightsails 9
Supplementary Figure 1. Example three-dimensional mesh surface construction 9
Supplementary Figure 2. Flow-chart diagram of flexible lightsail simulation code 11
Implementation of ray tracing for curved specular lightsails 12
Supplementary Figure 3. Ray-tracing plots for three example simulation frames 13
Simulating the dynamics of rigid lightsails 14
Time-stepping approach for numerical integration 17

Supplementary Note 4: Thermal modelling for curved Si lightsails 18
Radiative heat transfer 18
Supplementary Figure 4. Geometric factors for radiative heat transfer 19
Supplementary Figure 5. Comparison of simulation results 21
Thermal runaway 21
Supplementary Figure 6. Absorption coefficient for Si vs. temperature 22
Supplementary Figure 7. Simulation results for thermal runaway 23

Supplementary Note 5: Paraboloid Si_3N_4 lightsail not destabilized by secondary reflections 24
Supplementary Figure 8. Simulation results for the acceleration of a Si_3N_4 paraboloid lightsail 24

Supplementary Note 6: Stability analysis of metagrating designs 25

Supplementary Note 7: Calculating optical forces for metagratings 30

Supplementary Note 8: Alternative design for yaw-restoring torque 31
Supplementary Figure 9. Composite metagrating design for self-restoring torque about z-axis 31

Supplementary Note 9: Example passively stabilized metagrating lightsail w/ initial translation 33
Supplementary Figure 10. Acceleration dynamics of lightsails w/ initial translation 34

Supplementary Note 10: Deviation from flatness of flexible spinning lightsails 35
Supplementary Figure 11. Deviation from flatness of flexible spinning lightsail 35
Supplementary Figure 12. Maximum displacement of flexible spinning lightsail 36

Supplementary Note 11: Temperature & strain analysis for stabilized metagrating lightsail 37
Supplementary Figure 13. Peak, average and minimum lightsail temperature versus time 37
Supplementary Figure 14. Maximum strain 38

Supplementary Note 12: Effect of temperature on dynamics of flexible lightsails 39
Supplementary Figure 15. Comparison of x–y trajectories with and without absorption 40

Supplementary Note 13: Angular deviations of flexible lightsails from a flat shape 41
Supplementary Figure 16. Pitch θ and roll ϕ distribution...41
Supplementary Figure 17. Maximum angular deviations...42

Supplementary Note 14: Unstable cases of propelled flexible metagrating-based lightsails 43
Supplementary Figure 18. Unstable propulsion cases...44

Supplementary Note 15: Flexible vs. rigid metagrating sails at lower spin frequencies 45
Supplementary Figure 19. Comparison of flexible and rigid lightsail dynamics 45

Supplementary Note 16: Acceleration performance of flexible metagrating-based lightsail 46

Supplementary References...47

Supplementary Note 1: Discussion of Materials' Properties

We have collected a number of candidate material property values from the literature for the purpose of simulating the structural dynamics of lightsails. These appear in Supplementary Table 1 below. This is not intended as an exhaustive list or ranking of candidate materials for the interstellar lightsail, and importantly, it should be noted that the published properties of these materials can vary greatly depending on the method of fabrication, as well as the test geometry and method of measurement. Furthermore, most properties are reported based on room-temperature measurements, whereas during acceleration, lightsails will operate at elevated temperatures where material properties have been less comprehensively studied. We did not attempt to model temperature-dependent mechanical properties in the present study, although it would be straightforward to add this capability in the future. Experimental characterization of candidate lightsail material(s), as fabricated at relevant scale, and over the intended operating temperature range, will be required to draw conclusions about the viability of any specific lightsail design.

Aluminum and polyimide are typical materials used for solar sails; we include them as a point of comparison. Note that the stringent requirement of ultralow optical absorption precludes the use of even the most reflective of metals for interstellar lightsail application. It also seems unlikely that polymers could be used structurally in this application, owing to their low strength and limited temperature range. Other materials offering exceptional mechanical strength such as graphene and carbon nanotubes can likely be ruled out owing to their high optical absorption. However, there are likely a wide range of dielectrics and wide-bandgap semiconductors that may prove useful in lightsail applications, in addition to those shown in Supplementary Table 1.

For materials such as crystalline silicon, SiO_2 and diamond, the highest recorded strengths have been achieved by small (< 50 μm diameter) filaments of high-purity materials with pristine surfaces, tested in bending over a small mandrel to further limit the stressed surface area and thus the chances of encountering a surface defect which could initiate fracture. Achieving such high strengths in large-scale membrane geometries would pose considerable materials engineering and fabrication challenges. Furthermore, crystalline materials, whether bulk or 2D, may exhibit reduced strength if used to fabricate arbitrarily curved lightsail surfaces such as spheres, cones, or paraboloids, owing to relative weakness of certain crystal planes, or the inability to perfectly join crystal surfaces at domain boundaries.

Supplementary Table 1. Mechanical and thermal properties for candidate lightsail materials at room temperature.

Material	Structure type	Poisson ratio ν	Young's Modulus E [GPa]	Tensile strength σ_T [GPa]	Density ρ [g·cm^{-3}]	Compressive strength σ_C [GPa]	Thermal conductivity κ [W·m^{-1}K^{-1}]	Linear CTE α_L [ppm·K^{-1}]	Heat capacity C [J·g^{-1}K^{-1}]
**Silicon**[1–3]	Crystal filament	0.06–0.28	130–190	Up to 4.9	2.33	3.2	160	2.3–4.5	0.67
(111) surf.[4,5]	Crystal plane	0.26	169	2.1	2.33				
CVD poly[6]	Thin film	0.22	169	1.2	2.33				
**Diamond**[7,8]	Crystal filament	0.10–0.29	910–1250	Up to 7.5	3.52	9–16	2000–2100	0.8–4.8 (1.0 typ.)	0.51
CVD poly[9]	Thin film	0.2	1050	0.41					
CVD nano[10]	Thin film	0.03	750	5.0	3.27				
CVD UNCD[10]	Thin film	0.20	460	1.8	2.92				
**SiO_2**									
Fused silica[11]	Bulk	0.16	73	0.054	2.20	1.14	1.4	0.57	0.77
Quartz[12,13]	Crystal plane	0.16	97	0.165	2.65	2	10.7	7.1	0.71
Tempered glass[14,15]	Thick film	0.22	77	Up to 1.0	2.40		1.2	7.25	0.76
Silica fibers[16]	Filament			Up to 6.0					
**Si_3N_4** (LPCVD)[17–20]	Thin film	0.27	270	6.4	2.7	1–5	3	2.3	0.8
**MoS_2**[21–24]									
Single-layer	2D crystal plane	0.27	270	22	5.02		30–100	5	0.39
Multi-layer	2D crystal plane		200–330	21			80–110		
**Aluminum** (7075)	Bulk	0.33	72	0.50	2.80		130	24	0.96
**Polyimide**[25]	Thick film	0.34	2.5	0.069	1.42		0.12	20	1.10

Supplementary Note 2: Stationary burst diameter figure-of-merit

The stationary burst diameter figure of merit (D_{SB}) presented in the manuscript is useful for describing the relative area scale at which the candidate materials could be used to form perimeter-supported lightsails, and avoids making assumptions beyond the membrane's thickness and the effective pressure exerted against it. Our work is motivated by the Breakthrough Starshot concept, based on which we select notional parameters for the lightsail, including 0.1 g/m^2 areal mass and 10 GW/m^2 propulsion laser intensity. Developing functional lightsails is a complex engineering challenge in which these parameters are variables that must be optimized alongside many others, but these values are useful starting points for evaluating candidate membrane materials.

However, D_{SB} is conservative, and generally underestimates the practical area that could be spanned, for two primary reasons. Most obviously, the stationary constraint precludes acceleration and free flight of the lightsail—a lightsail intended for launch must have a lightweight support structure, which would accelerate along with the lightsail. Intuition suggests that a lightsail with extremely lightweight perimeter supports could span significantly larger areas, particularly if the beam is relatively uniform across the its area. In fact, the burst diameter becomes infinite if one assumes uniform illumination and zero structural mass. This is why we selected D_{SB} as our figure of merit, as it provides the conservative endpoint of this continuum, and avoids making assumptions about structural mass or rigidity.

This leads to the second issue regarding D_{SB}: the assumption of uniform beam pressure. As discussed in the manuscript, for the launch of interstellar lightsails, economic and practical considerations will likely necessitate that the propulsion beam operates at the diffraction limit, at least for the terminal phase of acceleration. Thus, designing lightsails for Gaussian beams is of practical relevance. Even in the stationary perimeter constraint case, the burst diameter of a membrane will be larger if the beam tapers off in intensity near the edge of the lightsail, assuming the same peak intensity at the beam center.

Perimeter supports are one of several options for imparting structural stability to lightsails in flight. The stationary burst diameter D_{SB} is a helpful figure of merit for comparing the relative strengths of candidate materials to function as perimeter-supported membranes for lightsail applications, but should not be taken as a limiting diameter for lightsail construction. Evaluating designs for viability will require one to identify and analyze specific materials and structures to provide the necessary support, and the solution may draw from combination of approaches (e.g. perimeter support and spinning).

To provide some first-order insights into how the maximum allowable lightsail diameter might differ from the D_{SB} figure of merit, we employ the mesh simulator to analyze the specific example of a Si lightsail, for which D_{SB} was calculated to be 1.09 m. We assume a 43 nm thick (0.1 g/m^2), flat circular membrane of this diameter, attached at its perimeter to a rigid hoop. In the first case, the hoop is stationary; in the second case, the hoop is assumed to have mass equal to that of the encircled membrane (0.095 g) and is constrained in all degrees of freedom except motion along the acceleration axis, thus allowing the support hoop to accelerate with the lightsail (note that we call this "free flying" despite the imposed constraints, which are necessary for successful simulation). The simulated geometry and excitation beam are aligned and symmetric about the acceleration axis. We use the mechanical properties of Si listed in Table 1. As this discussion pertains to mechanical (rather than optical or thermal) properties, we assume 100% reflectance, no absorption, and no emission, such that the temperature remains at 300 K and thermal stress does not occur. The beam intensity is slowly ramped up in the time domain until tensile failure is detected, for uniform illumination and Gaussian beams of various beam waist diameters (relative to the lightsail diameter). The results are summarized in Supplementary Table 2.

Supplementary Table 2. Mesh simulator burst-intensity results for a non-spinning, perimeter-supported Si membrane of D_{SB} diameter (1.09 m) and 43 nm (0.1 g/m^2) thickness, with uniform and variously sized Gaussian-beam illumination.

Gaussian illumination conditions				Stationary rigid hoop		Free-flying rigid hoop ($m_{hoop} = m_{sail}$)		
Beam size relative to lightsail $\frac{w_0}{r_s}$	Intensity at lightsail edge $\frac{I(r_s)}{I_0}$ (%)	Beam capture efficiency (%)	Beam uniformity efficiency (%)	Rupture I_0 (GW/m^2)	Relative force vs. 10 GW/m^2 uniform illumination (%)	Rupture I_0 (GW/m^2)	Rel. to 0.1 g/m^2 membrane at 10 GW/m^2 uniform illumination: Force (%)	Rel. to 0.1 g/m^2 membrane at 10 GW/m^2 uniform illumination: Acceleration (%)
∞ (uniform)	100	0	100	9.1	91	17.0	170	85
2	60.7	39.3	78.6	10.3	81	17.5	140	69
$\sqrt{2}$	36.8	63.2	63.2	11.6	63	18.0	114	57
1	13.5	86.5	43.3	14.2	61	19.8	85	43
$\sqrt{2}/2$	1.8	98.2	24.6	19.0	47	23.5	58	29

Simulations assume 100% reflectance and T = 300 K. w_0 is the Gaussian beam waist radius. r_s is the lightsail radius. I is the beam intensity (I_0 at the center).

While the accuracy of the time-domain simulation tool is somewhat limited compared to analytic solutions or finite-element solvers, it is adequate for this illustration. We note the simulated stationary burst intensity of 9.1 GW/m^2 for uniform illumination, whereas according to the analytical calculations used for D_{SB}, the burst intensity (pressure) should be 10.0 GW/m^2 (67 Pa). This should be kept in mind when interpreting the results shown in Supplementary Table 2—percentage values for force and acceleration are scaled to I_0 = 10.0 GW/m^2 rather than the simulated stationary burst intensity. As summarized in the table, assuming either finite Gaussian (versus uniform) illumination, or a finite-mass free-flying (versus stationary) perimeter support, permits the peak incident power intensity I_0 to exceed 10 GW/m^2 without rupturing the membrane. Thus, the membrane could be made larger, or operated under higher peak power intensities, than suggested by the D_{SB} figure of merit. Assuming the latter, we calculate the force imparted to the membrane at this elevated maximum intensity, relative to the uniform 10 GW/m^2 case that was assumed in calculating D_{SB}. For the free-flying rigid-hoop case, we also present the acceleration, relative to a theoretical free-flying membrane with no additional structural mass accelerating under uniform 10 GW/m^2 illumination. The acceleration of the hoop-supported structure is halved due to the assumption $m_{hoop} = m_{sail}$, which doubles the mass to be accelerated.

These cursory simulations yield the burst intensity for a lightsail of fixed diameter D_{SB}, rather than the burst diameter for the fixed I_0 value of 10 GW/m^2 established at the outset of the discussion. Although we do not quantify specific burst diameter values at this intensity for comparison to D_{SB}, it follows that a higher burst intensity for a certain lightsail diameter should equate to a larger burst diameter at a lower intensity. Ultimately, the maximal propulsion intensity will likely be limited by the components and materials of the lightsail spacecraft, in which case a near-uniform beam of this intensity would produce maximal acceleration. However, achieving high beam uniformity across the entire lightsail area throughout its acceleration would require a dramatically larger or less efficient, and thus costlier, laser propulsion system, and may pose additional challenges for beam-riding stability. These are trade-offs that must be optimized at the systems level, where structural stability of the lightsail is only one of many critical requirements.

Stability considerations and system-level implications

It is tempting to assume that the propulsion beam should be of uniform laser intensity, to minimize thermal gradients and force nonuniformities that could distort the lightsail shape. This after all is the operating regime for solar sails, which can navigate via active modulation of solar reflectance or other mechanisms[26–28]. However, producing uniform plane-wave illumination for relativistic interstellar lightsail propulsion would require a laser source of inconceivable power and size—the beam would of course be truncated to a top-hat profile, making its power needs tractable. Yet, focusing such a beam over the proposed acceleration distances would still require an impractically large transmitter area. The system would instead employ Goubau beam profiles, which are optimal for power transmission[29–31]. Such beam configurations were assumed in a recent system analysis and cost optimization of the Starshot mission concept[32]. These specific and optimal beam profiles are beyond the scope of our present work, but by noting more generally that the laser would likely be constructed no larger than necessary to achieve the target mission velocity, we conclude that the system should ideally be able to operate at or near the diffraction limit for the final phase of acceleration. We therefore restricted our study of beam-riding stability to static Gaussian beams, which reasonably approximate what can be expected from any optimized transmitter in this limit. Other profiles such as higher-order Gaussian, Goubau, or doughnut beams[33,34] may be favorable at earlier stages of acceleration when the propulsion system is less limited by diffraction, but we believe the challenge of lightsail acceleration under weakly focused Gaussian-like beam profiles is of somewhat fundamental importance to Starshot.

Closed-loop feedback trajectory control cannot be accomplished from the ground station: At the speed of light, the round-trip delay between an observation of the accelerating lightsail, and the arrival of a corrective adjustment of the laser source in an attempt to stabilize the trajectory via feedback control, would range up to several minutes at the end of the acceleration phase, whereas non-beam-riding lightsails can veer off course on a timescale of milliseconds. Additionally, atmospheric turbulence and practical technological limitations will cause at least some unpreventable perturbations to the desired beam position and profile during acceleration[35]. Thus, although some initial prescriptive corrective actions from the laser source may be feasible, the lightsail itself must ultimately be capable of aligning its acceleration trajectory to the beam axis, by passive or active means, without ground-based intervention. The challenge of steering the spacecraft then becomes primarily that of correctly pointing and slewing the direction of the ground-based laser source during acceleration. Although active optical control surfaces could in principle be employed on the lightsail to enhance beam-riding stability or adjust its final trajectory, developing such control surfaces to operate under the extreme beam intensities and mass constraints required for interstellar lightsails remains an unsolved challenge.

We thus conclude that beam-riding stability is a practical necessity for interstellar lightsail spacecraft, as have many others, leading to numerous lightsail designs and stability studies in recent literature, including this paper. The preliminary yet growing consensus is that passively beam-riding lightsails are feasible, based on increasingly practical designs and increasingly detailed calculations. These encouraging developments warrant a discussion of how passive beam-riding stability may impact the mission concept at the system level, from which one can identify key objectives for future work.

The most significant impact of beam-riding stability is a potential reduction in available acceleration distance. Passively stabilized lightsails must rely solely on the beam intensity gradient to produce the restoring forces and torques necessary for beam-riding, but this gradient will weaken as distances approach the diffraction limit. For a given lightsail, there is a limiting Gaussian beam diameter, and therefore a limiting acceleration distance, beyond which its passive beam-riding behavior will become inadequate to overcome the instantaneous or cumulative effects of perturbations, at which point it would escape the beam and be launched irrecoverably off course. This establishes a practical upper limit for the acceleration distance and thus the achievable velocity for a given system configuration. Critically,

this may occur at a substantially shorter distance than that over which the laser system can still focus a reasonable fraction of its energy onto the lightsail area.

For example, a spin-stabilized metagrating-based lightsail simulated in this study exhibited marginal beam-riding stability from a certain initial offset (perturbation) for a beam width of $w = 0.4D$, but flew off-beam from the same initial offset when the beam width was $w = 0.5D$—in which case the beam still focused over 99% of its power on the lightsail envelope as initially offset. It should be noted that this design did not attempt to optimize this or other aspects of acceleration performance, and should not suggest that such narrow beams are necessary for lightsail propulsion. Our present work should be considered primarily as an example of the techniques and methods necessary to begin the more rigorous design of optimally stabilized lightsails suitable for interstellar propulsion. However, this clearly illustrates that beam-riding constraints can limit acceleration distance, which makes it important to consider in future system-level optimizations. In particular, with respect to Gaussian beams of increasing diameter, it is important to analyze whether the result is a total disappearance of marginal stability, or simply a reduction in the trough width for oscillatory stability. In the latter case, usable acceleration distance will be limited by practical perturbations and their cumulative effect on lightsail misalignment, whereas in the former case, this provides a more fundamental limit on the usable acceleration distance.

Another important issue resulting from beam-riding stability constraints pertains to early-stage acceleration, during which the beam profile is less limited by diffraction. The Goubau configuration provides optimal overall power delivery to the lightsail area in this regime, becoming similar to a top-hat profile near the transmitter. Although we have not considered such profiles here, it is clear that perturbations would cause some amount of misalignment or distortion of the beam, and that beam-riding will require the lightsail to respond to the resulting gradient in beam intensity to produce restoring forces and torques.

Considering the nominal top-hat case, with the beam perfectly sized to the lightsail, any misalignment would result in a lune-shaped peripheral region of the lightsail falling outside of the beam. Presumably, the lightsail's design would ensure that this asymmetry (i.e., effective beam gradient) would produce a restorative (beam-riding) response. However, unless damping is provided, oscillatory motion would persist for the remainder of acceleration—this is an important issue for future work as discussed in the manuscript. However, and in any case, it is important to note that the misalignment results in a loss of propulsive efficiency, as some of the beam misses the lightsail. This is a fundamental necessity for passive beam-riding stability, and should be taken into account in system-level design and optimization. Quantifying this effect will require detailed consideration of the nature of perturbations, which would likely also be considered in the lightsail's design for stability. Further complicating matters is that the maximal permissible beam intensity may be limited due to thermal limits of the lightsail. It seems possible that rigorous optimization may result in a specific beam profile that differs from the nominal Goubau profile, which may be less efficient in the case of perfect alignment, but more effective over the entire acceleration sequence under during which the perturbations are likely to occur.

To conclude, we offer the following recommendations for future efforts to develop passive beam-riding lightsails: First, to determine the practical limitations of acceleration distance, that weakly focused (i.e., Gaussian-like) beams be considered, and that the maximal stable beam diameter be quantified or discussed in a practical context pertaining to transmitter power and size, distance, and expected perturbations. Second, to address earlier acceleration performance, that specific (e.g. Goubau-like) beams be considered and optimized, both for the purpose of analyzing lightsail beam-riding and thermal stability, but also for quantifying the impacts on propulsive efficiency likely to result from perturbations. These efforts will allow better understanding of the interstellar lightsail concept at the system level.

Supplementary Note 3: Mesh-based simulator for flexible lightsails

We have developed a time-domain simulator for studying the dynamic behavior of flexible lightsails being accelerated by optical forces. This is facilitated by modelling the lightsail as a discrete mesh, wherein the nodes represent mass, inertia, temperature, and shape; the edges represent the stiffness and thermal conductivity of the material; and enclosed triangles represent the surface area through which light interacts with the lightsail.

This code has been open-sourced at: https://github.com/Starshot-Lightsail

It is provided as a collection of scripts, functions, and data files for the MATLAB environment, which facilitates convenient access to all calculations and variables for analysis, plotting, or augmentation of functionality. Examples, documentation, and extensive commenting are provided to assist in its usage.

Simulations start with generation of the mesh. Nodes are assigned initial positions along the desired surface profile and meshed via Delaunay triangulation, with their spacing chosen to yield approximately uniform edge length and aspect ratio among the triangles. An example simulation mesh for a paraboloid lightsail is plotted in Supplementary Fig. 1. As of February 2024, the mesh generator supports simple geometries having circular, hexagonal, or square shape; flat, conical, parabolic, or spherical vertical profile, and revolved or faceted profile filling. Any number of materials can be defined; four parametric mappers are provided for assigning specific materials and optical surfaces throughout the mesh, based on which the elemental mechanical, thermal, and optical responses are computed.

The simulation process is outlined in Supplementary Fig. 2. Briefly, the evolution of the position, velocity, and temperature of the mesh is calculated iteratively in the time domain, using a fixed time step chosen to be substantially smaller than any vibrational modes of the mesh (typically, $1/20^{th}$ to $1/10^{th}$ of the period of the highest resonant frequency). Simulations begin at rest. If desired, a spin-up phase smoothly accelerates the lightsail to a specified spin-stabilization frequency, then the initial offsets in position and tilt are applied. This is followed by propulsion phases, include ramp-up of laser intensity, acceleration, and ramp-down, with optional coast periods prior to ramp-up and following ramp-down to enable observation of initial or residual dynamics. The propulsion beam may be defined parametrically based on lateral position (x, y), time, and downrange distance z; here, we employ a stationary Gaussian beam of fixed waist radius and peak intensity. The simulator produces various output plots summarizing the lightsail behavior, including a video animation, and records numerous state variables at specified intervals in monitor buffers, which are saved to file and remain in memory for subsequent analysis following completion of the simulation.

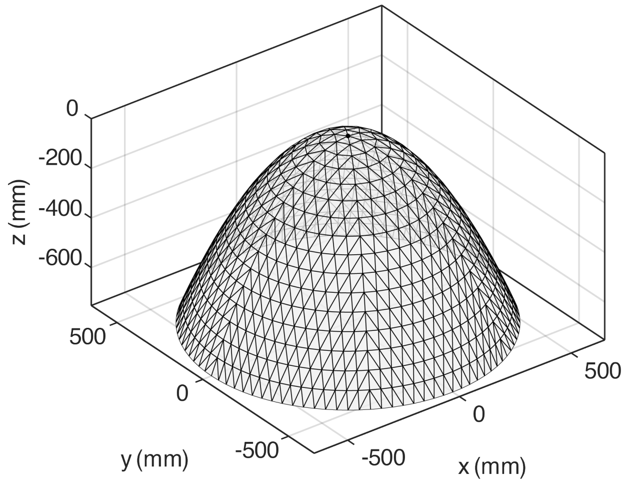

Supplementary Figure 1. Example three-dimensional mesh surface construction using Delaunay triangulation to model a paraboloid lightsail.

We employ a mass-spring model to approximate linear elastic behavior of flexible membranes, implicitly neglecting bending stiffness. Although not featured in this manuscript, solid-body dynamics are also supported, simultaneously, for any number of bodies comprising unique subsets of the mesh, thus including fully-rigid lightsails, flexible lightsails with rigid frames, subframes or crossmembers, or flexible lightsails containing rigid regions to approximate payloads. A tethered payload is also supported, implemented as a single point mass connected by a multitude of multi-element filaments to the lightsail perimeter (the payload and tethers are not subjected to optical or thermal interactions).

We implement several methods for calculating optical forces and absorption, including fixed reflectance and absorptance values, angle-dependent thin-film specular optics (transfer matrix method), ray tracing for specular non-planar geometries, and tabulated 2D data for non-specular surfaces such as metagratings (derived from experimental measurements or electromagnetic simulations). Beam occlusion is not modeled; thus lightsail shapes are limited to those having non-overlapping envelopes when projected in the illumination plane. Polarization is partially supported, but cannot vary relative to the lightsail; further development is needed to model arbitrary polarization, and for ray tracing, to account for its change upon reflection.

Thermal behavior includes heat conduction, radiative cooling, radiative heat transfer, linearized thermal expansion, and parametric temperature-dependent optical absorption. The heating, cooling, and optical forces calculated at each triangular element are distributed to the adjoining nodes, which represent the temperature distribution of the structure. Thermal conduction is calculated along the mesh edges based on the local material properties and mesh geometry, whereas temperature is calculated at each node based on its mass and the specific heat of the material. As the temperature distribution is known throughout the structure, the effects of linear thermal expansion, which contributes to thermal strain, can be analyzed.

Mechanical or thermal failure of the membrane is detected when the stress exceeds the material's tensile strength or the temperature exceeds its specified limit, respectively. The simulation can then be terminated, or allowed to proceed to determine the margin by which the chosen conditions will exceed the material capabilities. Alternately, to enable cursory depictions of the progression of such failures, we can delete the affected elements from the ongoing simulation at their moment of failure; however, this is not intended to accurately model the dynamics of tensile or thermal failures. Specifically, our simulator does not model collisions between the collapsed lightsail elements, neglects beam occlusion effects for inverted shapes, and is not suited to model fracture propagation or thermal decomposition. Thus, collapsed and tattered lightsails are not simulated very accurately, but this approach can be helpful for illustrative purposes.

Although the simulator provides useful insights into the behavior of flexible lightsails, it is based on numerical approximations and employs numerous simplifying assumptions in its methods. Most notably to the context of this manuscript, the present approach cannot be used to study the scenario of polarization mismatch, which is necessary to evaluate whether our spinning lightsails are stable in non-rotating beams, or whether lightsails can self-synchronize their rotation to that of the beam during acceleration (see further discussion in Supplementary Note 8). These and other limiting assumptions could be addressed by employing more comprehensive physics models in the future. In addition to full polarization support, other topics might include non-isotropic properties or nonlinear behaviors of materials, or additional temperature-dependent effects. Ultimately, as a time-domain technique, our simulator cannot calculate steady-state behavior, and due the accumulation of numerical errors introduced by explicit time stepping, simulations cannot be performed over indefinite timescales to definitively prove marginal stability. Typical acceleration durations of up to 1–10 seconds were simulated in this work, which is considerably longer than the timescale of the instabilities addressed here, but shorter than the proposed acceleration time (of up to several minutes) envisioned by the Starshot mission concept. Simulations of shorter duration can nonetheless useful in analyzing stability at various key phases of acceleration, by selecting appropriate beam profile and wavelength for each.

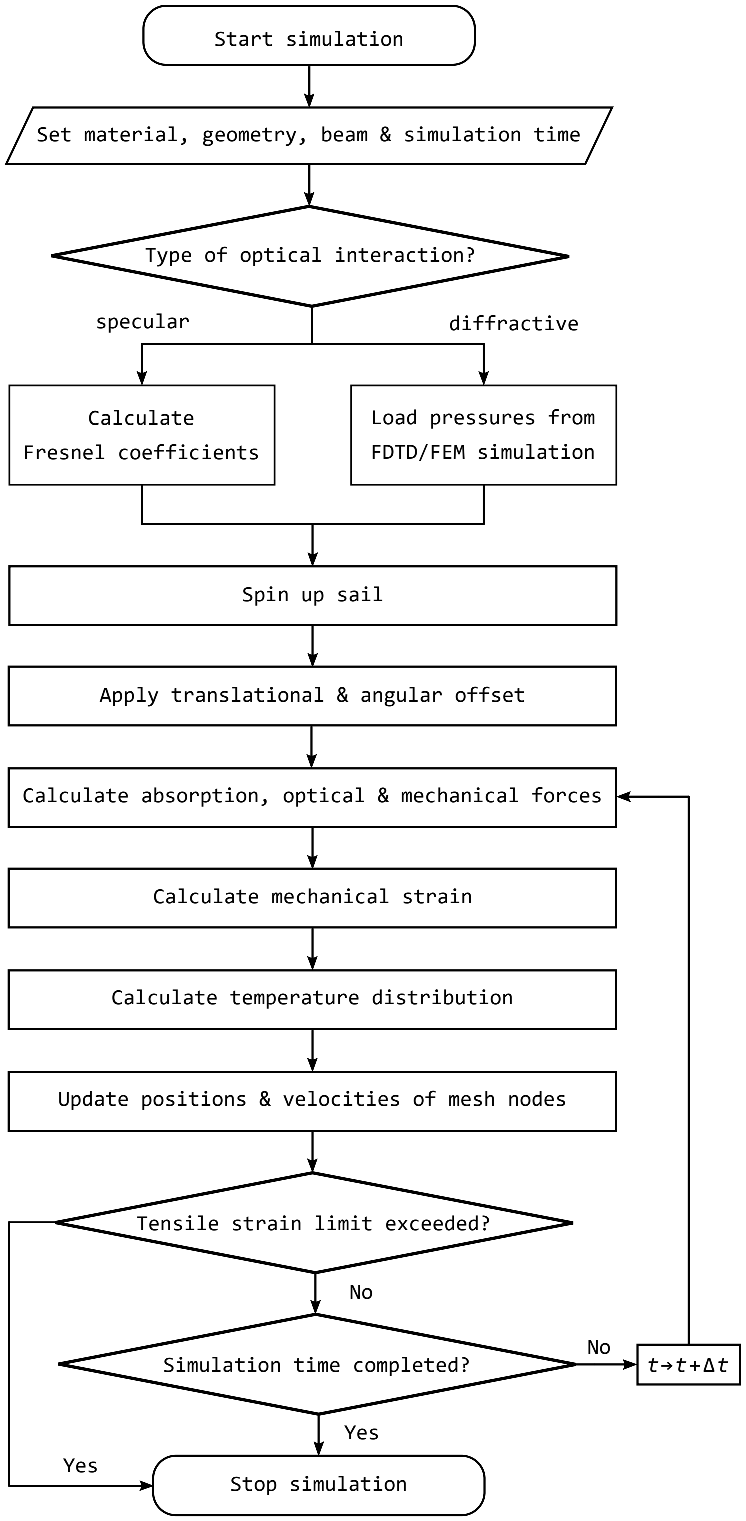

Supplementary Figure 2. Flow-chart diagram of flexible lightsail simulation code

Implementation of ray tracing for curved specular lightsails

Our initial efforts to model lightsail structural dynamics considered propulsive forces resulting from the incident beam interaction only, calculating heating due to absorption, and thrust due to the change in momentum of the light being reflected. This approach is inadequate for curved lightsails, in which light reflected by this initial interaction would impinge upon other parts of the lightsail, imparting additional optical force and absorptive heating. We term these and subsequent interactions as *secondary reflections*. To determine the effects of secondary reflections on the behavior of curved lightsails, we implemented a ray-tracing approach, summarized here.

Like all other computations in our simulations, ray-tracing calculations are performed discretely. Source rays originate from the initial interaction of the propulsion beam with each triangle, at the triangle centroids, with direction determined by reflection of the incident beam angle, and with power determined by the local beam intensity multiplied by the projected triangle area and attenuated by the angle-dependent specular reflectance of the local membrane surface. To determine the reflectance and absorption values, we use Fresnel coefficients using the transfer matrix method to account for thin-film effects, and to accelerate computation, we employ lookup tables rather than direct calculations during simulation. Rays are traced to their point of intersection with the nearest next triangle, if available. For successful rays, we compute the resulting optical force and absorption at this point of intersection, based on incidence angle, surface normal, and local optical properties, then compute the resulting power and direction of the reflected ray originating at this point. The process is repeated until all rays are geometrically exhausted or are culled for lack of remaining power, or upon reaching an iteration cutoff threshold chosen to prevent needless propagation of rays circulating along curved surfaces at glancing incidence (we typically limit ray-tracing depth to six, but for the structures that we have studied, all substantially discernible effects of secondary rays are captured with just two iterations).

The ray-tracing results (forces, absorption) are summed and applied to each triangle, with forces assumed to act upon the triangle at its centroid, regardless of where the incident rays arrived. This is a modest simplification, which speeds up calculation and has negligible impact on accuracy for a sufficiently fine mesh. However, due to the faceted nature of the mesh and the discrete nature of this ray-tracing approach, diverging reflections from curved surfaces sometimes produce mild quantization artifacts in the temperature profiles when ray tracing is enabled, as can be seen in Fig. 4 and Supplementary Figs. 3, 5, and 8. Light reflected from any single mesh element imparts effect to at most one other element, even if the underlying surface curvature represented by the source element would cause its reflected light to diverge and impinge an area represented by multiple receiver elements. However, we believe that the approach is reasonable and appropriate for our modelling to date, as it conserves energy and produces consistent results in all regards (except for subtle artifact patterns in temperature distributions) over a wide range of mesh densities.

Presently, ray tracing can be used with specular membranes only, using either constant or angle-dependent optical properties, but it is incompatible with the metasurface (2D lookup table) surface model. In principle, the method could be extended to consider the multiple rays generated by such diffractive surfaces, but this is not warranted in our case, as the metasurface elements are designed to stabilize flat lightsails, where secondary reflections do not occur.

The raytracing calculations are computationally laborious compared to other simulation steps, which run reasonably quickly as vector or matrix operations on our computing environment (MATLAB). We were unable to implement ray tracing with speeds practical for our simulations within the base MATLAB environment. Transitioning the simulator to a compiled language might provide suitable if not better overall speeds, but we instead elected to employ the MATLAB's Parallel Computing Toolbox to perform the ray tracing on a GPU, allowing ray-tracing simulations to proceed at reasonable speeds on our desktop PCs. Unfortunately, for the presently released code version, this limits the availability of ray tracing to users who have a compatible graphics card and license to this additional toolbox.

Supplementary Fig. 3 shows renderings of exemplary ray-tracing calculations. To make the rays discernible, we produced these plots for extremely coarse meshes, comprising only 600 triangles, whereas simulations presented elsewhere in the manuscript used meshes of ~3000–10,000 triangles.

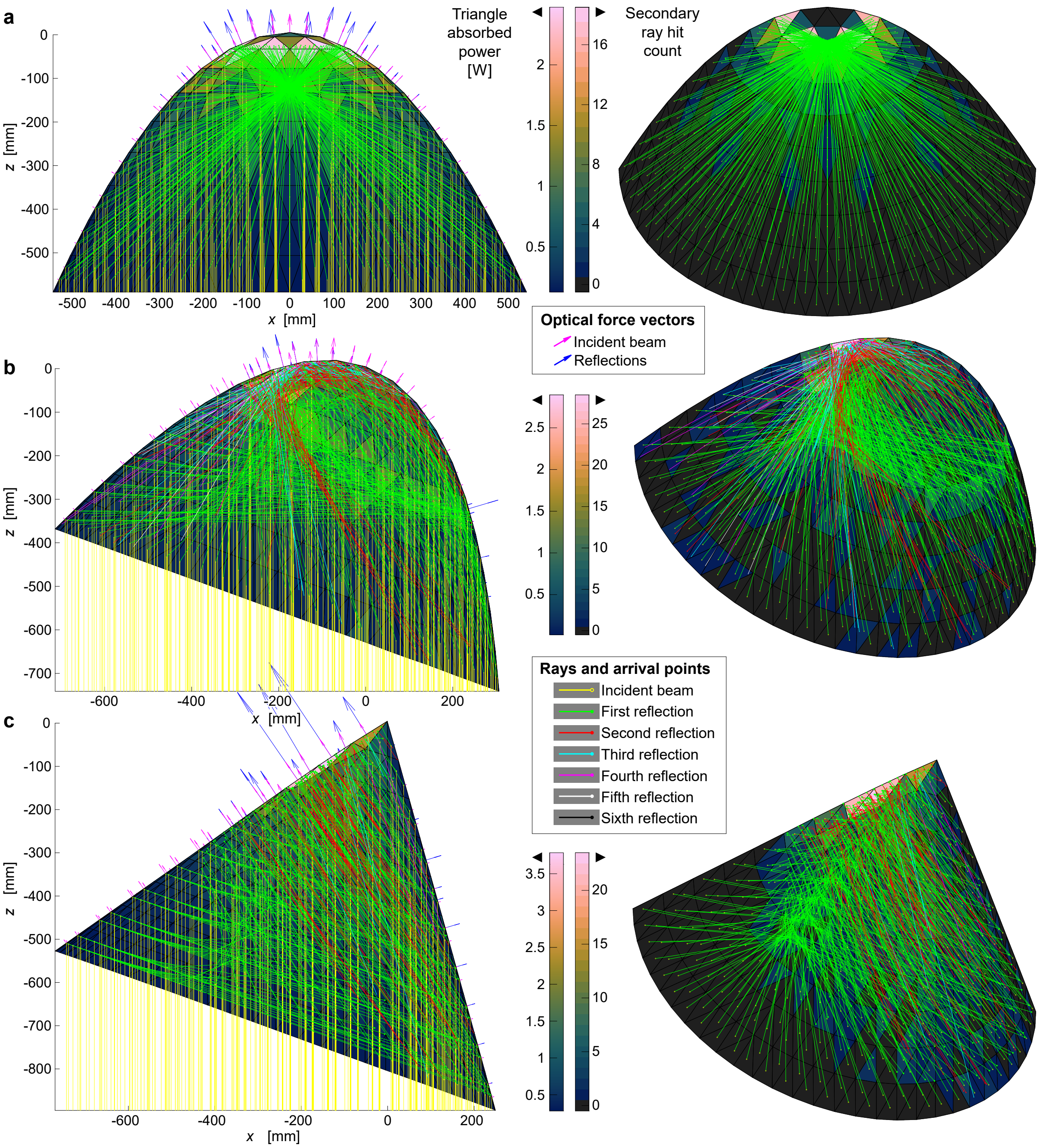

Supplementary Figure 3. Raytracing plots for three example simulation frames: **(a)** a paraboloid lightsail, aligned and centered on the beam, producing 576 secondary reflection rays; **(b)** the same paraboloid lightsail, tilted 20° to the beam (1025 reflection rays); and **(c)** a conical lightsail of similar aspect ratio, tilted 20° to the beam (865 reflections). Simulations assume optical properties for 43 nm-thick Si as discussed above, and Gaussian illumination with $I_0 = 5$ GW/m^2 and $w_0 = \sqrt{2}\, r_{\mathrm{sail}}$. All plots show a 3D rendering the lightsail and ray traces, bisected in the *x*-*z* plane through the lightsail vertex point. Plots on the left also show the implied incident beam rays (yellow), and the resulting force vectors acting upon each triangle (force vectors are rendered from triangle centroids in 3D and are mostly obscured by the lightsail, but visible near the edge of the envelope). In the plots on the right, the rendering view angle is rotated 45° to better illustrate the 3D distribution of rays.

Simulating the dynamics of rigid lightsails

Numerical simulations of rigid-body dynamics are based on previously reported methods[36]. Motion of a metagrating-based rigid silicon nitride lightsail is captured by its center of mass position $\mathbf{r} = (x, y, z)$ and angular orientation given by the Euler angles $\boldsymbol{\alpha} = (\psi, \theta, \phi)$. While $\mathbf{r}$ describes translational motion within the inertial lab frame I, $\boldsymbol{\alpha}$ denotes rotation of the lightsail or equivalently the lightsail's body frame B with respect to I. By adopting the 1-2-3 or *x-y'-z''* rotation sequence, transformation from the inertial frame to the body frame is described the direction cosine matrix

$$\mathbf{H}_I^B(\psi,\theta,\phi) = \mathbf{R}_z(\psi)\mathbf{R}_y(\theta)\mathbf{R}_x(\phi) = \begin{pmatrix} c_1c_2 & c_1s_2s_3 + s_1c_3 & -c_1s_2c_3 + s_1s_3 \\ -s_1c_2 & -s_1s_2s_3 + c_1c_3 & s_1s_2c_3 + c_1s_3 \\ s_2 & -c_2s_3 & c_2c_3 \end{pmatrix},$$

with $c_i = \cos(\alpha_i)\,, s_i = \sin(\alpha_i)$, $\alpha_1 = \psi, \alpha_2 = \theta$ and $\alpha_3 = \phi$, and single-axis rotation matrices

$$\mathbf{R}_x(\phi) = \begin{pmatrix} 1 & 0 & 0 \\ 0 & \cos(\phi) & \sin(\phi) \\ 0 & -\sin(\phi) & \cos(\phi) \end{pmatrix}, \mathbf{R}_y(\theta) = \begin{pmatrix} \cos(\theta) & 0 & -\sin(\theta) \\ 0 & 1 & 0 \\ \sin(\theta) & 0 & \cos(\theta) \end{pmatrix},$$

$$\mathbf{R}_z(\psi) = \begin{pmatrix} \cos(\psi) & \sin(\psi) & 0 \\ -\sin(\psi) & \cos(\psi) & 0 \\ 0 & 0 & 1 \end{pmatrix}.$$

Consequently, transformation from the body frame to the inertial frame can be mathematically described by the inverse or transpose of the orthogonal direction cosine matrix

$$\mathbf{H}_B^I(\boldsymbol{\alpha}) = \left(\mathbf{H}_I^B(\boldsymbol{\alpha})\right)^T.$$

When simulating the angle-dependent optical pressures $\mathbf{p}_i(\boldsymbol{\alpha})$ on each of the four regions or sectors of the lightsail in the body frame, the electric field vector is assumed to be perfectly aligned with the y-axis of the body frame at all times. This assumption results in the optically induced pressure on each region j of the lightsail being independent of ψ, i.e., $\mathbf{p}_i(\boldsymbol{\alpha}) = \mathbf{p}_i(\theta, \phi)$. Consequently, in the body frame, the polarization vector in the four regions will be given by

$$\mathbf{E}_i = \mathbf{H}_I^B(\beta_j, \theta, \phi)\mathbf{E}_I,$$

where $\mathbf{E}_I = E_0(0, 1, 0)$ and $\beta_{1,2,3,4} = 0, \pi, \pi/2, 3\pi/2$.

With the laser beam propagation direction being along the negative z-direction, $\mathbf{k}_I = k_0(0, 0, -1)$, the wavevector for each region j is given by

$$\mathbf{k}_i = \mathbf{H}_I^B(\beta_j, \theta, \phi)\mathbf{k}_I,$$

from which the incidence angle can be calculated as $\arctan(k_{x,i}/k_{z,i})$.

Due to the mirror-symmetric design of the lightsail and the permanent alignment of the electric field vector with the body frame y-axis, only regions 1 and 3 need to be simulated. In COMSOL Multiphysics, 2D electromagnetic models of region 1 (TE metagrating) and region 3 (TM metagrating) were set up by applying periodic (Floquet) boundary conditions to the boundaries of the TE and TM unit cell. By integrating the Maxwell Stress tensor, one obtains the optically induced pressures on regions 1 and 3 in their respective local frame $\mathbf{p}'_{\mathrm{TE}}(\theta, \phi) = \mathbf{p}'_1(\boldsymbol{\alpha})$ and $\mathbf{p}'_{\mathrm{TM}}(\boldsymbol{\alpha}) = \mathbf{p}'_3(\boldsymbol{\alpha})$, respectively. These pressures need to be transformed to the body frame of the lightsail via

$$\mathbf{p}_i(\theta,\phi)=\begin{bmatrix}\cos(\beta_i) & -\sin(\beta_i) & 0\\ \sin(\beta_i) & \cos(\beta_i) & 0\\ 0 & 0 & 1\end{bmatrix}\mathbf{p}_i'(\theta,\phi),$$

where we can take advantage of the following relationships due to the mirror-symmetric design and assumption of $\psi = 0$:

$$\mathbf{p}_2'(\theta,\phi)=\mathbf{p}_1'(-\theta,-\phi),$$

$$\mathbf{p}_4'(\theta,\phi)=\mathbf{p}_3'(-\theta,-\phi).$$

Finally, we note that $p_{1,y}' \ll p_{1,x}', p_{1,z}'$ and $p_{3,y}' \ll p_{3,x}', p_{3,z}'$, i.e., the out-of-plane pressure components in our 2D COMSOL simulations are more than two orders of magnitude smaller than the other two components. For this reason, we ignore the out-of-plane pressure components obtained from the Maxwell stress tensor integration in our analysis, thus setting $p_{1,y}' = p_{3,y}' = 0$.

Having simulated the angle-dependent optical pressures $\mathbf{p}_i(\boldsymbol{\alpha})$ in the body frame, we can then calculate the optically induced forces in the inertial frame, assuming Gaussian spatial variation of the beam intensity, as

$$\mathbf{F}(\mathbf{r},\boldsymbol{\alpha})=\mathbf{H}_B^I(\boldsymbol{\alpha})\sum_i \mathbf{F}_i(\mathbf{r},\boldsymbol{\alpha}),$$

$$\mathbf{F}_i(\mathbf{r},\boldsymbol{\alpha})=\eta\iint r d\phi dr\,\mathbf{p}_i(\theta,\phi)\frac{I_0}{c}\exp\left(-\frac{2}{w^2}\left\|\begin{bmatrix}x\\ y\\ 0\end{bmatrix}+\mathbf{H}_B^I(\boldsymbol{\alpha})\begin{bmatrix}r\cos(\varphi)\\ r\sin(\varphi)\\ 0\end{bmatrix}\right\|^2\right),$$

with the projected area $\eta = \cos(\theta)\cos(\phi)$, and the following integration limits for respective lightsail region given by

$$r_{1,2,3,4}\in\left[0,\frac{D}{2}\right],\varphi_1\in\left[-\frac{\pi}{6},\frac{\pi}{6}\right),\varphi_2\in\left[\frac{\pi}{6},\frac{5\pi}{6}\right),\varphi_3\in\left[\frac{5\pi}{6},\frac{7\pi}{6}\right),\varphi_4\in\left[\frac{7\pi}{6},\frac{11\pi}{6}\right).$$

Optically induced torques can be similarly expressed as

$$\boldsymbol{\tau}(\mathbf{r},\boldsymbol{\alpha})=\sum_i \boldsymbol{\tau}_i(\mathbf{r},\boldsymbol{\alpha}),$$

$$\boldsymbol{\tau}_i(\mathbf{r},\boldsymbol{\alpha})=\eta\iint r d\phi dr\begin{bmatrix}r\cos(\varphi)\\ r\sin(\varphi)\\ 0\end{bmatrix}\times\mathbf{p}_i(\theta,\phi)\frac{I_0}{c}\exp\left(-\frac{2}{w^2}\left\|\begin{bmatrix}x\\ y\\ 0\end{bmatrix}+\mathbf{H}_B^I(\boldsymbol{\alpha})\begin{bmatrix}r\cos(\varphi)\\ r\sin(\varphi)\\ 0\end{bmatrix}\right\|^2\right).$$

The total force $\mathbf{F}(\mathbf{r},\boldsymbol{\alpha})$ and torque $\boldsymbol{\tau}_i(\mathbf{r},\boldsymbol{\alpha})$ serve as inputs to the twelve equations of motion that fully describe kinematics and dynamics of a rigid lightsail given by

$$\dot{\mathbf{r}}=\mathbf{v},$$

$$\dot{\mathbf{v}}\approx\frac{1}{m}\left(\mathbf{H}_I^B(\boldsymbol{\alpha})\right)^T\mathbf{F}(\mathbf{r},\boldsymbol{\alpha}),$$

$$\dot{\boldsymbol{\alpha}}=\begin{bmatrix}\dot{\psi}\\ \dot{\theta}\\ \dot{\phi}\end{bmatrix}=\begin{bmatrix}-\cos(\psi)\tan(\theta) & \sin(\psi)\tan(\theta) & 1\\ \sin(\psi) & \cos(\psi) & 0\\ \cos(\psi)\sec(\theta) & -\sin(\psi)\sec(\theta) & 0\end{bmatrix}\begin{bmatrix}\omega_x\\ \omega_y\\ \omega_z\end{bmatrix}=\mathbf{L}_B^I\boldsymbol{\omega},$$

$$I_x \dot{\omega}_x = \left(I_y - I_z\right)\omega_y \omega_z + \tau_x,$$

$$I_y \dot{\omega}_y = (I_z - I_x)\omega_x \omega_z + \tau_y,$$

$$I_z \dot{\omega}_z = \left(I_x - I_y\right)\omega_x \omega_y + \tau_z.$$

The beam is assumed to be constant along z, the gravitational term is omitted due to propulsion in the radiation-pressure dominated regime, and the Euler angle rates are related to the angular velocity $\boldsymbol{\omega}$ by the orthogonal matrix $\mathbf{L}_B^I$.

The principal moments of inertia I_x, I_y, I_z for our round lightsail design can be derived as follows

$$I_x = \chi^{\mathrm{TE}}\left(\int_{-\pi/6}^{\pi/6}\int_0^{D/2} r^2 \sin^2(\varphi)\, r dr d\varphi + \int_{5\pi/6}^{7\pi/6}\int_0^{D/2} r^2 \sin^2(\varphi)\, r dr d\varphi\right) + \chi^{\mathrm{TM}}\left(\int_{\pi/6}^{5\pi/6}\int_0^{D/2} r^2 \sin^2(\varphi)\, r dr d\varphi + \int_{7\pi/6}^{11\pi/6}\int_0^{D/2} r^2 \sin^2(\varphi)\, r dr d\varphi\right),$$

$$I_y = \chi^{\mathrm{TE}}\left(\int_{-\pi/6}^{\pi/6}\int_0^{D/2} r^2 \cos^2(\varphi)\, r dr d\varphi + \int_{5\pi/6}^{7\pi/6}\int_0^{D/2} r^2 \cos^2(\varphi)\, r dr d\varphi\right) + \chi^{\mathrm{TM}}\left(\int_{\pi/6}^{5\pi/6}\int_0^{D/2} r^2 \cos^2(\varphi)\, r dr d\varphi + \int_{7\pi/6}^{11\pi/6}\int_0^{D/2} r^2 \cos^2(\varphi)\, r dr d\varphi\right),$$

$$I_z = I_x + I_y,$$

where χ^{TE} and χ^{TM} are the mass per unit area of the TE and TM region given by

$$\chi^i = \rho\left(t + h\frac{w_1^i + w_2^i}{d^i}\right), i \in \{\mathrm{TE}, \mathrm{TM}\},$$

with ρ = 2700 kg m^{-3} being the density of silicon nitride, and the TE and TM unit cells being geometrically defined by w_1^{TE} = 600 nm, w_1^{TM} = 520 nm, $w_2^{\mathrm{TE/TM}}$ = 200 nm, d^{TE} = 1600 nm, d^{TM} = 1350 nm. Hence, numerical values for χ^{TE} and χ^{TM} are given by 1.08 x 10^{-3} kg m^{-2} and 1.12 x 10^{-3} kg m^{-2}, respectively.

Knowing the mass per unit area of the TE and TM region, we can also calculate the total mass of the meter-sized round lightsail, i.e., with diameter D = 1 m, as

$$m = \pi\left(\frac{\chi^{\mathrm{TE}}}{3} + \frac{2\chi^{\mathrm{TM}}}{3}\right)\left(\frac{D}{2}\right)^2 \approx 0.867 \text{ g}.$$

We can express all equations and quantities in terms of normalized units, starting with unitless lengths $\mathbf{r}' = \mathbf{r}/D$ and unitless time $t' = t/t_0$ by defining

$$t_0 = \sqrt{\frac{mc_0}{I_0 D}},$$

where c_0 is the speed of light and I_0 the peak intensity of the incident Gaussian beam, which in our case was chosen to be I_0 = 1 GW m^{-2}. As a result, the spinning frequency f_z can also be normalized via

$$\omega_z' = 2\pi f_z t_0.$$

Finally, we can also express forces and torques as unitless quantities with $\mathbf{F}' = \mathbf{F}/(I_0 D^2/c_0)$ and $\boldsymbol{\tau}' = \boldsymbol{\tau}/(I_0 D^3/c_0)$ as shown in Fig. 5 in the main text.

With the previously introduced equations of motion, we can numerically evolve the equations of motion by expressing them as a vectorial differential equation of first order

$$\frac{d\mathbf{u}'}{dt} = \dot{\mathbf{u}}' = \mathbf{f}(\mathbf{u}'),\ \mathbf{u}' = (\mathbf{r}', \mathbf{v}', \boldsymbol{\alpha}, \boldsymbol{\omega}') = \left(x', y', z', v_x', v_y', v_z', \psi, \theta, \phi, \omega_x', \omega_y', \omega_z'\right),$$

which can be numerically evolved using MATLAB's ode45 solver for given initial conditions $(\mathbf{r}_0', \mathbf{v}_0', \boldsymbol{\alpha}_0, \boldsymbol{\omega}_0')$. From here on, we drop the primed notation indicating that variables are dimensionless for better readability.

Time-stepping approach for numerical integration

In the simplest approach, the dynamics of flexible lightsails comprises interconnected nodes with positions $\mathbf{r}_n$ and velocities $\mathbf{v}_n$ can be numerically evolved via

$$\mathbf{v}_n(t_i + \Delta t) = \mathbf{v}_n(t_i) + \frac{\mathbf{F}_n(t_i)}{m_n}\Delta t, \qquad \mathbf{r}_n(t_i + \Delta t) = \mathbf{r}_n(t_i) + \mathbf{v}_n(t_i + \Delta t)\Delta t,$$

Which corresponds to the symplectic or semi-explicit/implicit Euler method of first order. Better numerical accuracy can be achieved with higher-order integration methods such as the Runge-Kutta method used to simulate the flexible lightsail reported in Fig. 6:

$$\mathbf{u}_n(t_i) = \begin{pmatrix} \mathbf{r}_n(t_i) \\ \mathbf{v}_n(t_i) \end{pmatrix} \to \mathbf{u}_n(t_i + \Delta t) = \mathbf{u}_n(t_i) + \frac{\Delta t}{6}\left(\mathbf{k}_{1,n} + 2\mathbf{k}_{2,n} + 2\mathbf{k}_{3,n} + \mathbf{k}_{4,n}\right),$$

$$\mathbf{k}_{1,n} = \begin{pmatrix} \mathbf{v}_n(t_i) \\ \mathbf{F}_n\big(t_i, \mathbf{r}_n(t_i)\big)/m_n \end{pmatrix}, \qquad \mathbf{k}_{2,n} = \begin{pmatrix} \mathbf{v}_n(t_i) + \frac{\Delta t}{2}\frac{\mathbf{F}_n\big(t_i, \mathbf{r}_n(t_i)\big)}{m_n} \\ \mathbf{F}_n\left(t_i + \frac{\Delta t}{2}, \mathbf{r}_n(t_i) + \frac{\Delta t}{2}\mathbf{v}_n(t_i)\right)/m_n \end{pmatrix},$$

$$\mathbf{k}_{3,n} = \begin{pmatrix} \mathbf{v}_n(t_i) + \frac{\Delta t}{2}\left(\mathbf{F}_n\left(t_i + \frac{\Delta t}{2}, \mathbf{r}_n(t_i) + \frac{\Delta t}{2}\mathbf{v}_n(t_i)\right)/m_n\right) \\ \mathbf{F}_n\left(t_i + \frac{\Delta t}{2}, \mathbf{r}_n(t_i) + \frac{\Delta t}{2}\left(\mathbf{v}_n(t_i) + \frac{\Delta t}{2}\frac{\mathbf{F}_n\big(t_i, \mathbf{r}_n(t_i)\big)}{m_n}\right)\right)/m_n \end{pmatrix},$$

$$\mathbf{k}_{4,n} = \begin{pmatrix} \mathbf{v}_n(t_i) + \mathbf{F}_n\left(t_i + \frac{\Delta t}{2}, \mathbf{r}_n(t_i) + \frac{\Delta t}{2}\left(\mathbf{v}_n(t_i) + \frac{\Delta t}{2}\frac{\mathbf{F}_n\big(t_i, \mathbf{r}_n(t_i)\big)}{m_n}\right)\right)/m_n \\ \mathbf{F}_n\left(t_i + \Delta t, \mathbf{r}_n(t_i) + \frac{\Delta t}{2}\left(\mathbf{v}_n(t_i) + \frac{\Delta t}{2}\left(\mathbf{F}_n\left(t_i + \frac{\Delta t}{2}, \mathbf{r}_n(t_i) + \frac{\Delta t}{2}\mathbf{v}_n(t_i)\right)/m_n\right)\right)\right)/m_n \end{pmatrix},$$

for node n with corresponding force $\mathbf{F}_n$ acting on it at a specific time step and position.

Supplementary Note 4: Thermal modelling for curved Si lightsails

During review of the manuscript, we were asked how radiative heat transfer might affect the lightsail temperature. Earlier efforts had considered only the effects of optical absorption (heating), radiative cooling, and thermal conduction within the membranes. For the flat lightsails, radiative heat transfer can be ignored, but it may affect the temperature distribution in the curved lightsails. To address this, we implemented a radiative heat transfer model in our simulator, and employed it to quantify the effects of radiative heat transfer on the simulated temperatures of a parabolic Si lightsail.

To calculate radiative heat transfer, we construct an $n \times n$ view factor matrix V during mesh generation:

$$V(i,j) = \frac{A_j}{{d_{ij}}^2} \cos(\theta_{ij}) \cos(\theta_{ji}),$$

where n is the number of triangles comprising the mesh, A_j is the area of triangle j, d_{ij} is the distance between triangles i and j, and θ_{ij} is the view angle towards triangle j at the surface of triangle i (relative to the surface normal). The calculation is discrete, evaluated at and between triangle centroid coordinates.

The radiative conductance matrix K further includes the area and emissivity ε of the emitting triangles, and the absorption of the receptor triangles, which under our assumptions is equal to emissivity via Kirchhoff's law:

$$K(i,j) = A_i \varepsilon_i \varepsilon_j \cdot V(i,j).$$

Generally speaking, the emission from a source triangle i varies with temperature and as a function of angle and wavelength, and similarly, the absorption of this radiation at triangle j depends on the receiving material's spectral and angular absorption, weighted by the source spectrum. Here, we simply assume constant scalar hemispherical emissivity with Lambertian angular distribution, which is adequate for first-order calculations. Additionally, the view factors will change as the geometry evolves during simulation. It is straightforward to recalculate the radiative conductance matrix throughout simulations, but it is computationally costly, and for lightsails that are stable in beam-riding, the geometric changes over time are negligible. Thus, we retain the radiative heat transfer matrix calculated during mesh generation and use it throughout the simulation duration.

Radiative heat transfer is calculated during the simulations as:

$$Q_{\mathrm{RHT}}(i,j) = k_B T_i^4 K(i,j).$$

Row-wise summation yields the power radiated by triangle i towards all other triangles; column-wise summation yields the power absorbed by triangle j from all other triangles. We calculate only the latter—which yields the radiative heating of all triangles—because the former is already accounted for in the calculation of radiative cooling, as:

$$Q_{\mathrm{EMS}}(i) = k_B T_i^4 \left(\varepsilon_i^{\mathrm{in}} + \varepsilon_i^{\mathrm{out}}\right),$$

where $\varepsilon_i^{\mathrm{in}}$ is the emissivity and absorptivity value for inward-facing surfaces (and thus the value used for radiative heat transfer above), and $\varepsilon_i^{\mathrm{out}}$ is the emissivity value for outward-facing surfaces. We generally assume the same emissivity value for both surfaces, which is the case for simple membranes, and have not yet performed optical design efforts or modelling of high-emissivity photonic structures, which could give rise to asymmetric emissivity. The curved lightsail shapes studied to date have simple convex geometries in which radiative transfer occurs without occlusion and between inner surfaces only, thus we did not need to compute occlusion or bifacial radiative heat transfer. More complex geometries would require more rigorous approaches in modelling.

Supplementary Fig. 4 shows the results of view factor computation for several convex lightsails, including the specific geometry from Fig. 3 and Fig. 4 of the manuscript as well as the geometry from the discussion below. Free-space obscuration is the summation of emission view factors, and indicates the fraction of thermal radiation from each triangle that impinges other lightsail surfaces. Illumination factor is the summation of absorption view factors, and indicates the geometric factor of radiative heating. Both are normalized for the inner hemisphere only. Note that our calculation is performed discretely, evaluated at the triangle centroid coordinates, rather than integrating over surface areas. This apparently overestimates the view factor for adjoining triangles at corners or faceted edges, as shown in Supplementary Fig. 4c and 4d. However, the discrete approximation is reasonable for the smoothly curved lightsails presented in the manuscript.

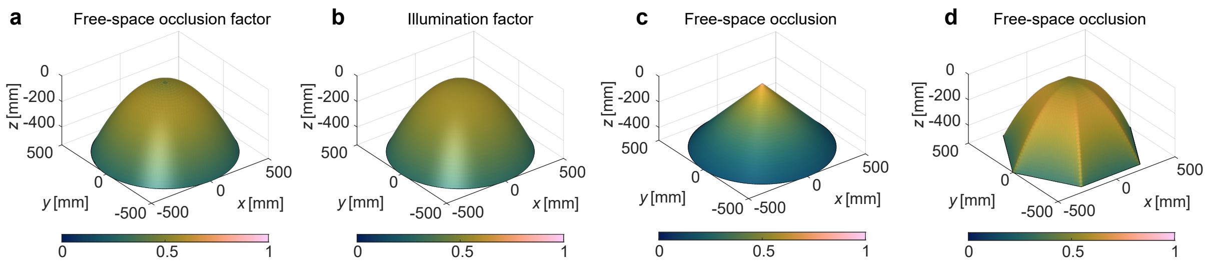

Supplementary Figure 4. Geometric factors for radiative heat transfer. **(a)** Free-space obscuration and **(b)** radiative illumination factors for the paraboloid lightsails of Fig. 3 and Fig. 4, and view factors for an example **(c)** conical and **(d)** hexagonally faceted paraboloid lightsail.

We evaluated radiative heat transfer for the specific lightsail scenario depicted in Fig. 4 of the manuscript—a 1 m-diameter Si paraboloid of 43 nm thickness. A limitation of our work on curved lightsails is that we have not considered the specific photonic designs necessary to achieve high-emissivity radiative cooling of the lightsail. Such photonic designs have been extensively investigated for various material systems[37–40]. In our prior studies on the structural and beam-riding behavior of flexible lightsails, we have simply assumed a fixed emissivity value for radiative cooling, and focused our attention on determining the membranes' optical properties only at the propulsion wavelength. For specular surfaces, we calculate the angle-dependent reflectance and absorption values using Fresnel coefficients, based on literature-reported complex refractive indices. Applying this approach to thermal infrared wavelengths and integrating across the Planck blackbody spectrum yields extremely low emissivity values for 43 nm-thick Si, on the order of 1×10^{-5}–2×10^{-5} over the 300–600 K range. Despite the exceptionally low optical absorption value for the Si membrane (~3×10^{-10} at normal incidence, $\lambda = 1.55$ µm, and $T = 300$ K), this emissivity is inadequate to prevent overheating of the lightsail. Only a modest improvement in emissivity would be needed to bring about reasonable equilibrium temperatures, but we believe that higher emissivity may be achievable through photonic designs, and that this will be of practical necessity for lightsail spacecraft that must eventually accommodate imperfections and incorporate other functional materials—all of which must still have extremely low absorption, but which could introduce considerably more absorption than pure crystalline Si. Thus, for our work on curved Si lightsails, we assume that highly effective radiative cooling will be achieved by incorporating photonic enhancements of yet-unknown design on the lightsail surface, producing an assumed emissivity of 0.1, and seek to similarly modify the assumed absorption of the membrane to account for any additional absorption caused by this emissive surface (not speculating on the mass or other effective membrane property changes associated with the emissive surface for this first-order analysis).

A key challenge facing radiative cooling structures for lightsails, in addition to mass restrictions, is that they must not substantially absorb power from the propulsion beam, which is a somewhat contradictory objective to achieving high emissivity, particularly at elevated temperatures. Here, to approximate the maximal allowable absorption resulting from the emissive structures (as well as any other functional materials or imperfections present), we assume a higher absorption coefficient for the lightsail material, 1×10^{-2} cm^{-1}, which is more than two orders of magnitude above that expected for room-temperature Si (~4×10^{-5} cm^{-1}). It should be noted that the absorption of Si increases strongly with temperature, reaching the value assumed here at approximately 520 K. As described by Jaffe et al.[41], the increasing absorption coefficient of Si leads to thermal runaway at temperatures in this temperature range (depending on emissivity and incident power density). Thus, we have essentially assumed the largest permissible absorption coefficient for the lightsail membrane, as well as an optimistic but perhaps achievable value for effective emissivity that could result from an optimized photonic patterning of emissive materials. It remains to be determined whether such a low-absorption lightweight emissive surface could be realized for this application; we believe our assumptions to be near the practical limit for Si lightsails.

Applying this radiative heat transfer analysis to the paraboloid Si lightsails from Fig. 3 and Fig. 4 yields several insights. First, the magnitude of radiative self-heating is somewhat negligible compared to the magnitude of absorptive heating and radiative cooling, especially that resulting from the focused secondary reflections of the incident beam, which dramatically increase the local temperature. Supplementary Fig. 5 shows the results of radiative heat transfer simulations for an example scenario in which the secondary reflections lead to a peak temperature of ~650 K. Adding radiative heat transfer to the model increased the average lightsail temperature by up to ~4 K, although the peak maximum temperature was essentially unchanged (in a simulation without secondary reflections, the average temperature increase due to radiative heat transfer was ~2 K at the same simulation time). Note that the simulation shown in Supplementary Fig. 5 differs slightly from that presented in Fig. 4 (primarily in that we chose a lower spin frequency [90 Hz], which destabilized beam-riding). We found the effects of radiative heat transfer to be similar across other simulations, including those with prolonged steady-state beam-riding (≥ 1 s). For example, the 100 Hz spin-stabilized lightsail shown in Fig. 3 exhibited a steady-state 2.6 K increase in average temperature, and a 1.7 K increase in peak temperature, when we enabled radiative heat transfer in the model. Thus, radiative heat transfer appears to have little effect on the lightsail temperature, due in part to the relatively low assumed emissivity of 0.1, meaning that only 10% of the self-radiation is reabsorbed. Radiative heat transfer could be more prominent for lightsails with high emissivity, or geometries with higher view factors.

Interestingly, the effect of radiative heat transfer is dramatically greater than the effect of thermal conduction, which is quite negligible. Given the extreme aspect ratio of the simulated structures, this is not surprising, even though considerable efforts were previously invested to ensure thermal conduction was correctly modelled in our simulations. Thermal conductivity will likely play a critical role in localized cooling of the lightsail, for example, near payloads or defects, and is also of concern for propagation of thermal runaway resulting from dust impacts[42]. Successful lightsail spacecraft designs will likely require careful attention to thermal control, and require the use of high-conductivity materials such as diamond or MoS_2. However, at the macroscopic scale, it appears that thermal conduction is of little utility in cooling the lightsail through heat spreading, at least for Si membranes. It should be noted that the visualization of heat conduction in Supplementary Fig. 5 may be misleading: What is plotted is the net conductive heat flow into or out of each mesh node, divided by the adjoining surface area. This is appropriate for comparing the net effect of thermal conduction on lightsail temperature, on the same basis as the effects of optical absorption and thermal radiation shown in Supplementary Fig. 5g – 5j. However, it does not depict the magnitude of conductive heat flux within the membrane, which is considerably higher.

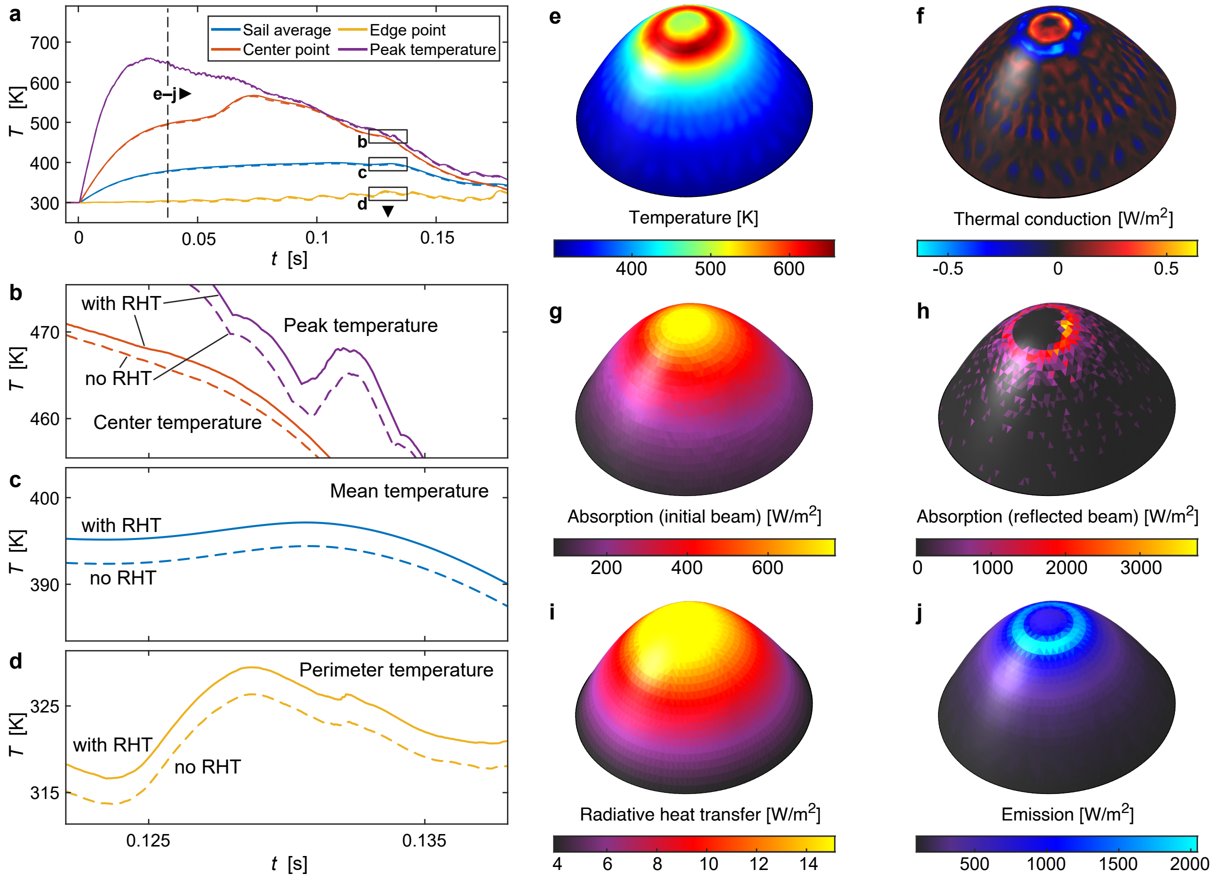

Supplementary Figure 5. Comparison of simulation results with and without the consideration of radiative heat transfer (RHT), for a Si paraboloid lightsail of the same geometry as presented in Fig. 3 and Fig. 4 (some conditions differ; see text). (**a** – **d**) show lightsail temperatures vs. time. (**e** – **j**) show the spatial distribution of relevant values throughout the mesh at $t = 36$ ms.

Thermal runaway

A final insight results from turning our attention to the issue of thermal runaway in Si. In our previous work on Si paraboloids, which did not take into account the effects of multiple reflections, we predicted peak equilibrium operating temperatures of ~500 K, which is slightly below the threshold for thermal runaway for our assumed emissivity. In fact, this was the rationale for our selection of the lightsail absorption coefficient, which is approximately that of Si at 520 K. However, considering the effects of multiple reflections, which for paraboloids become highly focused in certain regions, the peak simulated temperatures increased to 650 K. The localized beam intensity likely also exceeds the threshold for two-photon-absorption (TPA) initiated thermal runaway, which Holdman et al.[41] calculated to be ~5 GW/m^2 for Si used in a nanoresonator configuration—this is why we initially chose a peak beam intensity of 5 GW/m^2. We do not consider TPA in our present work, and do not know its limiting threshold intensity in this situation, but can conclude in general that focused reflections (caustics) from curved lightsails could be problematic for any materials with nonlinear absorption processes. In the simulated case, the lightsail reaches runaway temperatures even without TPA, and we do not include it in the model.

It is straightforward to implement a temperature-dependent absorption coefficient in our simulation code. Because the real refractive index of Si does not change substantially with temperature, the reflection and propulsive optical forces can be assumed to be independent of temperature. We do not propose or analyze the materials or design of a high-emissivity surface in this work, but suspect such would utilize SiO_2, Si_3N_4, or similar materials for their long-wavelength infrared absorption and relatively stable optical properties at elevated temperatures. Regardless of composition, one can conclude that emissivity will begin to decrease with increasing temperatures as the blackbody spectrum shifts from thermal infrared wavelengths (for which the emissive photonic texturing would be optimized) towards the near-infrared (where the lightsail must necessarily be non-absorbing). However, due to the broad spectral width of blackbody radiation, we expect the emissivity change to be insignificant over the temperature range in which runaway takes root, whereas the Si absorption coefficient increases exponentially with temperature, making the effects of temperature-dependent emissivity somewhat irrelevant once runaway begins. Thus, taking into account the temperature dependence of silicon's absorption coefficient alone is a reasonable first-order approach to study thermal runaway.

To study this example scenario, we fit a simple model for the Si absorption coefficient at $\lambda = 1.5$ μm, from 300 K to 1000 K, based on literature values[41,43]. The source data and model fit are shown in Supplementary Fig. 6. Our simulations assume a constant base absorption value, which is implemented as an effective absorption coefficient of 1×10^{-2} cm^{-1} for the material, to which we add the temperature-dependent Si absorption.

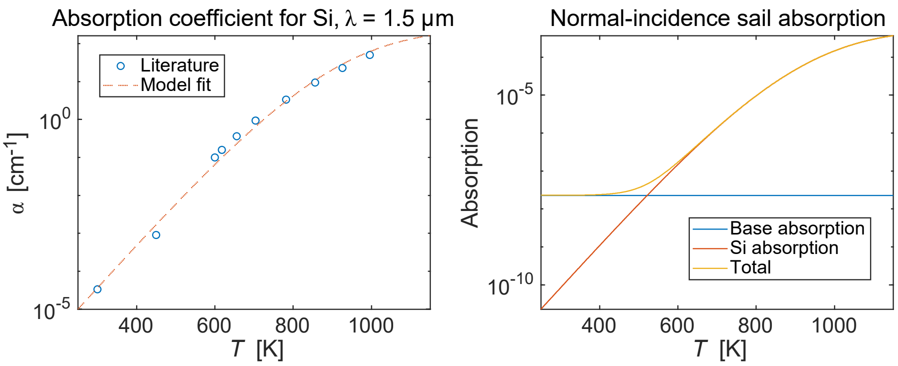

Supplementary Figure 6. Left: Absorption coefficient for Si vs. temperature, reported by Holdman et al.[41], and Rogne et al.[43], and model fit used for simulations. Right: Effective absorption vs. temperature used to simulate onset of thermal runaway.

The results of a simulation incorporating this model are shown in Supplementary Fig. 7, confirming that the Si paraboloid lightsails studied here would experience thermal runaway due to the focused reflections within the lightsail. We use the melting temperature of Si, ~1400 K, as the threshold temperature for mechanical failure. This is reached on a timescale of ~10 ms after the beam is turned on, although the dynamics is likely not well captured due to the coarse (centimeter-scale) size of the mesh elements, as well as the discrete nature of our simplified ray-tracing scheme, which may not accurately capture the peak intensity of the reflected caustics. Jaffe et al. model runaway with a submicron-scale finite element grid to elucidate the propagation of runaway, discussing in particular how thermal conduction can play a role in either propagating or arresting the spread of destructive runaway initiated by local hotspots[42]. Our simulator appears to show the thermal failure propagating quickly throughout the lightsail, but this is not primarily a result of thermal conduction; rather, it is an artifact resulting from how temperature and optical interactions are modelled by nodes and triangles of

the mesh. The propagation of thermal runaway would certainly involve thermal conduction at the local scale, but may also involve radiative heating or convection over larger length scales, the latter of which may deposit absorptive materials elsewhere on the lightsail or impart destructive mechanical impulse. Taking such effects into account is well beyond the scope of our simulation efforts.

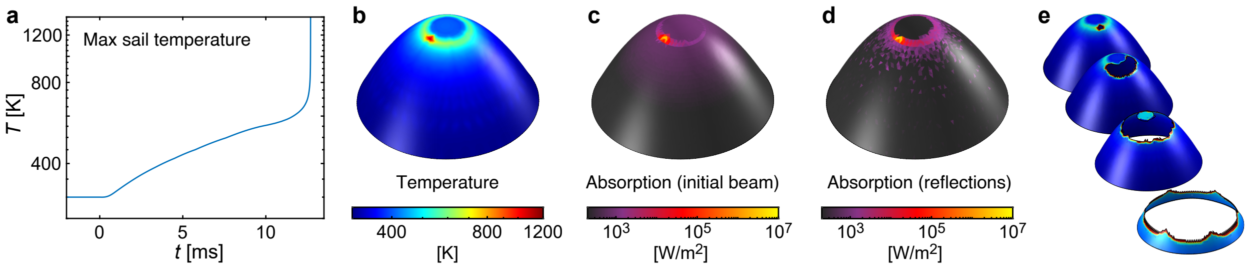

Supplementary Figure 7. Simulation results for thermal runaway in the Si paraboloid lightsail due to the temperature-dependent absorption of Si. (**a**) Maximum lightsail temperature vs. time. The beam ramps up to $I_0 = 5$ GW/m^2 over 1 ms, starting at $t = 0$ s. Overheating occurs at $t = 13$ ms. (**b–d**) Temperature, incident beam absorption, and reflected ray absorption profiles for the lightsail at $t = 13$ ms, immediately prior to the material exceeding its failure temperature. (**e**) Apparent propagation of thermal runaway in the simulator (see notes in text), plotted with the temperature colormap from panel **(b)**.

In any case, while arresting the propagation of localized thermal runaway is an important design challenge for lightsails, it is not particularly relevant in this case, because the runaway results from excessive light concentration intrinsic to the lightsail's shape rather than a point defect or transient condition. Thermal design may be a problematic challenge for convex lightsails, which experience multiple reflections, particularly if their shape concentrates the reflected light. This is in addition to the generally destabilizing propulsive effect of the secondary reflections discussed in the manuscript. However, curved lightsail designs cannot be ruled out. Viable curved lightsails include those with adequately low absorption and high emissivity, those whose shapes avoid highly focused secondary reflections, and those with relatively low specular reflectance, which would reduce the power contained in secondary reflections and thus the impact on trajectory and temperature. For example, Supplementary Fig. 8 shows simulation results for a Si_3N_4 paraboloid lightsail of the same shape and areal density ($t = 37$ nm) as the Si lightsail discussed above, where secondary reflections do not destabilize the beam-riding behavior, and where the focused heating due to secondary reflections is somewhat less pronounced (although we note in this case that the lightsail has inadequate cooling even without secondary reflections).

Supplementary Note 5: Paraboloid Si_3N_4 lightsail not destabilized by secondary reflections

To illustrate that not all curved lightsails are disrupted by secondary reflections, as we observed for the studied Si paraboloids, we present the following simulation of a Si_3N_4 paraboloid lightsail, whose structural and beam-riding stability is not disrupted by the addition of secondary reflections to the analysis. The geometry and simulation conditions are identical to the Si paraboloid of Fig. 4, except: (i) the initial lightsail offset to the beam is 0.14 times the lightsail radius (x_0, y_0 = 50 mm) rather than 0.10 (x_0 = 50 mm, y_0 = 0) due to an oversight; (ii) the membrane thickness is 37 nm to maintain 0.1 g/m^2 areal density; and (iii) all mechanical and optical properties are set to values appropriate for a Si_3N_4 membrane of this thickness, using a propulsion wavelength of λ = 1064 nm. We do not assume additional optical absorption or thermal emission; values are calculated from the Fresnel coefficients at the propulsion wavelength and over the 300 K blackbody spectrum, respectively. We acknowledge the inconsistency of using a fixed room-temperature emissivity value for a material whose simulated temperature reaches 1000 K. Based on the room-temperature complex refractive index spectra for Si_3N_4, emissivity would decrease dramatically at such temperatures, suggesting another pathway to thermal runaway. This is beyond the scope of our present study; the intent in presenting this simulation is to illustrate that secondary reflections do not destabilize beam-riding for all curved specular lightsails.

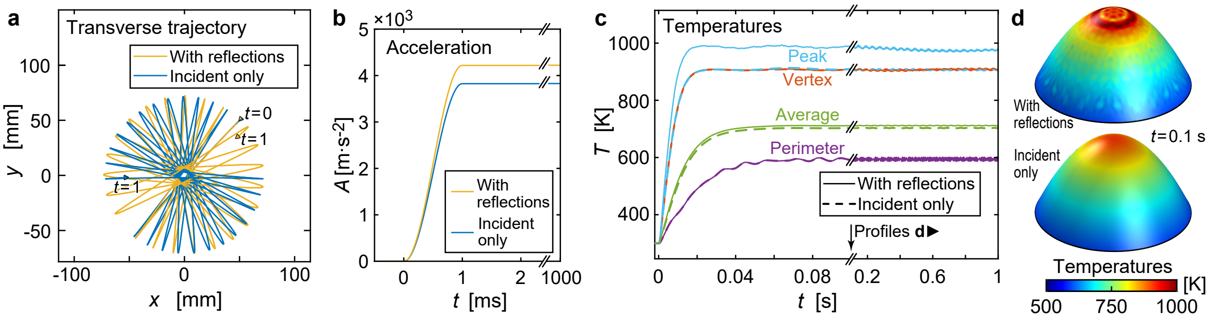

Supplementary Figure 8. Simulation results for the acceleration of a Si_3N_4 paraboloid lightsail, with vs. without secondary reflections, using the same geometry and simulation conditions as the Si lightsail depicted in Fig. 4, except as noted in the text. (**a**) *x*-*y* plane trajectory; both simulations produce the same amplitude of motion, with slightly different precession rates. (**b**) Acceleration vs. time; the impulse from secondary reflections is proportionally lower (compared to the Si lightsail) owing to the lower reflectance of the Si_3N_4 membrane. (**c**) Temperature monitors vs. time; the maximal temperature increase due to secondary reflections is ~80 K (1.09x) vs. ~150 K (1.25x) for the Si paraboloid. (**d**) Temperature profiles at t = 0.1 s, with and without secondary reflections. Simulations do not account for temperature-dependent emissivity, which may be more significant for the Si_3N_4 case (see discussion of thermal runaway in Supplementary Note 4). The patterning evident in the temperature profile for the "with reflections" simulation is due to quantization effects in ray tracing (see discussion in Supplementary Note 3).

Supplementary Note 6: Stability analysis of metagrating designs

For rigid lightsails, linear stability analysis can be performed to predict whether a given composite metagrating design is unstable. We stress that conclusions about stability cannot be drawn alone from linear stability analysis due to the inexistence of damping terms in the equations of motion; hence, numerical evolution of the differential equations is necessary to confirm marginal stability. Moreover, we only consider translational and rotational degrees of freedom in our stability analysis, as there is no stabilization mechanism in propulsion direction. Hence, we rewrite parts of the equations of motion as

$$\frac{d\tilde{\mathbf{u}}}{dt} = \dot{\tilde{\mathbf{u}}} = \tilde{\mathbf{f}}(\tilde{\mathbf{u}}),\ \tilde{\mathbf{u}} = (x, y, v_x, v_y, \psi, \theta, \phi, \omega_x, \omega_y, \omega_z), \tilde{\mathbf{f}} = (v_x, v_y, f_x, f_y, h_\psi, h_\theta, h_\phi, f_\phi, f_\theta, f_\psi).$$

In the case of a *non-spinning* rigid lightsail, we note that $\tilde{\mathbf{u}}_0 = \mathbf{0}$ is the trivial equilibrium of the system, which corresponds to the lightsail riding the beam. Consequently, the Jacobian is calculated as $\tilde{\mathbf{f}}'$ evaluated at $\tilde{\mathbf{u}}_0$

$$\tilde{\mathbf{f}}'(\tilde{\mathbf{u}}_0) = \begin{bmatrix} 0 & 0 & 1 & 0 & 0 & 0 & 0 & 0 & 0 & 0 \\ 0 & 0 & 0 & 1 & 0 & 0 & 0 & 0 & 0 & 0 \\ f_{xx} & f_{xy} & 0 & 0 & f_{x\psi} & f_{x\theta} & f_{x\phi} & 0 & 0 & 0 \\ f_{yx} & f_{yy} & 0 & 0 & f_{y\psi} & f_{y\theta} & f_{y\phi} & 0 & 0 & 0 \\ 0 & 0 & 0 & 0 & h_{\psi\psi} & h_{\psi\theta} & h_{\psi\phi} & h_{\psi\omega_x} & h_{\psi\omega_y} & h_{\psi\omega_z} \\ 0 & 0 & 0 & 0 & h_{\theta\psi} & h_{\theta\theta} & h_{\theta\phi} & h_{\theta\omega_x} & h_{\theta\omega_y} & h_{\theta\omega_z} \\ 0 & 0 & 0 & 0 & h_{\phi\psi} & h_{\phi\theta} & h_{\phi\phi} & h_{\phi\omega_x} & h_{\phi\omega_y} & h_{\phi\omega_z} \\ f_{\phi x} & f_{\phi y} & 0 & 0 & f_{\phi\psi} & f_{\phi\theta} & f_{\phi\phi} & f_{\phi\omega_x} & f_{\phi\omega_y} & f_{\phi\omega_z} \\ f_{\theta x} & f_{\theta y} & 0 & 0 & f_{\theta\psi} & f_{\theta\theta} & f_{\theta\phi} & f_{\theta\omega_x} & f_{\theta\omega_y} & f_{\theta\omega_z} \\ f_{\psi x} & f_{\psi y} & 0 & 0 & f_{\psi\psi} & f_{\psi\theta} & f_{\psi\phi} & f_{\psi\omega_x} & f_{\psi\omega_y} & f_{\psi\omega_z} \end{bmatrix}\Bigg|_{\tilde{\mathbf{u}}_0},$$

where we adopted the following notation for partial derivatives, $f_{ij} = \partial f_i/\partial j$.

In our case with only pitch- and roll-restoring behavior and translational stability, many of the matrix elements are either zero, very small and thus approximately zero, or can be calculated analytically, leaving us with a Jacobian $\hat{\mathbf{f}}'$ matrix of full rank that has a reduced dimension given by

$$\hat{\mathbf{f}}'(\tilde{\mathbf{u}}_0) \approx \begin{bmatrix} 0 & 0 & 1 & 0 & 0 & 0 & 0 & 0 \\ 0 & 0 & 0 & 1 & 0 & 0 & 0 & 0 \\ f_{xx}|_{\tilde{\mathbf{u}}_0} & 0 & 0 & 0 & f_{x\theta}|_{\tilde{\mathbf{u}}_0} & 0 & 0 & 0 \\ 0 & f_{yy}|_{\tilde{\mathbf{u}}_0} & 0 & 0 & 0 & f_{y\phi}|_{\tilde{\mathbf{u}}_0} & 0 & 0 \\ 0 & 0 & 0 & 0 & 0 & 0 & 0 & 1 \\ 0 & 0 & 0 & 0 & 0 & 0 & 1 & 0 \\ 0 & f_{\phi y}|_{\tilde{\mathbf{u}}_0} & 0 & 0 & 0 & f_{\phi\phi}|_{\tilde{\mathbf{u}}_0} & 0 & 0 \\ f_{\theta x}|_{\tilde{\mathbf{u}}_0} & 0 & 0 & 0 & f_{\theta\theta}|_{\tilde{\mathbf{u}}_0} & 0 & 0 & 0 \end{bmatrix}.$$

By numerically evaluating the remaining nonzero matrix elements of the Jacobian matrix, the presence of real parts in any of its eigenvalues indicates exponential growth of the respective solution to the equations of motion and thus instability of the laser-propelled system. Due to the lack of damping terms in the system's equations of motion, eigenvalues with real parts will always come in pairs of positive and negative real part.

The case of *spinning* rigid lightsails requires a more careful stability analysis, where the absolute values of the complex eigenvalues of the monodromy matrix, which can be obtained from numerical

integration involving the system's Jacobian matrix, determine whether spinning lightsails are stable or not. Importantly, ω_z is no longer assumed to be zero (or close to zero), but takes on a finite value, i.e., 2π times our desired spinning frequency, instead. Similarly, the yaw angle $\psi = \psi(t)$ will vary between 0 and 2π during a period of $2\pi/\omega_z$ and thus be time-dependent. To underline these differences, we evaluate $\tilde{\mathbf{f}}'(\tilde{\mathbf{u}}_0)$ with ω_z = constant and $\psi = \psi(t)$ to be

$$\tilde{\mathbf{f}}'(\tilde{\mathbf{u}}_0) = \begin{bmatrix} 0 & 0 & 1 & 0 & 0 & 0 & 0 & 0 & 0 & 0 \\ 0 & 0 & 0 & 1 & 0 & 0 & 0 & 0 & 0 & 0 \\ f_{xx}|_{\tilde{\mathbf{u}}_0} & f_{xy}|_{\tilde{\mathbf{u}}_0} & 0 & 0 & f_{x\psi}|_{\tilde{\mathbf{u}}_0} & f_{x\theta}|_{\tilde{\mathbf{u}}_0} & f_{x\phi}|_{\tilde{\mathbf{u}}_0} & 0 & 0 & 0 \\ f_{yx}|_{\tilde{\mathbf{u}}_0} & f_{yy}|_{\tilde{\mathbf{u}}_0} & 0 & 0 & f_{y\psi}|_{\tilde{\mathbf{u}}_0} & f_{y\theta}|_{\tilde{\mathbf{u}}_0} & f_{y\phi}|_{\tilde{\mathbf{u}}_0} & 0 & 0 & 0 \\ 0 & 0 & 0 & 0 & 0 & 0 & 0 & 0 & 0 & 1 \\ 0 & 0 & 0 & 0 & 0 & 0 & 0 & \sin(\psi(t)) & \cos(\psi(t)) & 0 \\ 0 & 0 & 0 & 0 & 0 & 0 & 0 & \cos(\psi(t)) & -\sin(\psi(t)) & 0 \\ f_{\phi x}|_{\tilde{\mathbf{u}}_0} & f_{\phi y}|_{\tilde{\mathbf{u}}_0} & 0 & 0 & f_{\phi\psi}|_{\tilde{\mathbf{u}}_0} & f_{\phi\theta}|_{\tilde{\mathbf{u}}_0} & f_{\phi\phi}|_{\tilde{\mathbf{u}}_0} & 0 & -\omega_z & 0 \\ f_{\theta x}|_{\tilde{\mathbf{u}}_0} & f_{\theta y}|_{\tilde{\mathbf{u}}_0} & 0 & 0 & f_{\theta\psi}|_{\tilde{\mathbf{u}}_0} & f_{\theta\theta}|_{\tilde{\mathbf{u}}_0} & f_{\theta\phi}|_{\tilde{\mathbf{u}}_0} & \omega_z & 0 & 0 \\ f_{\psi x}|_{\tilde{\mathbf{u}}_0} & f_{\psi y}|_{\tilde{\mathbf{u}}_0} & 0 & 0 & f_{\psi\psi}|_{\tilde{\mathbf{u}}_0} & f_{\psi\theta}|_{\tilde{\mathbf{u}}_0} & f_{\psi\phi}|_{\tilde{\mathbf{u}}_0} & 0 & 0 & 0 \end{bmatrix}.$$

To further simplify, we remind ourselves that $\tilde{\mathbf{f}}(\tilde{\mathbf{u}})$ can be linearly expanded around the "equilibrium" $\tilde{\mathbf{u}}_0 = (0, 0, 0, 0, \psi(t), 0, 0, 0, 0, \omega_z)$ as

$$\dot{\tilde{\mathbf{u}}} = \tilde{\mathbf{f}}(\tilde{\mathbf{u}}) \approx \tilde{\mathbf{f}}(\tilde{\mathbf{u}}_0) + \tilde{\mathbf{f}}'(\tilde{\mathbf{u}}_0)(\tilde{\mathbf{u}} - \tilde{\mathbf{u}}_0),$$

noting that

$$\tilde{\mathbf{f}}(\tilde{\mathbf{u}}_0) = [0 \quad 0 \quad 0 \quad 0 \quad \omega_z \quad 0 \quad 0 \quad 0 \quad 0 \quad 0]^T,$$

which means that $\tilde{\mathbf{u}}_0$ is not a true equilibrium. Nevertheless, evaluating the second term on the right-hand side of the Taylor-expanded equation above yields

$$\tilde{\mathbf{f}}'(\tilde{\mathbf{u}}_0)(\tilde{\mathbf{u}} - \tilde{\mathbf{u}}_0) = \tilde{\mathbf{f}}'(\tilde{\mathbf{u}}_0)\tilde{\mathbf{u}} - \tilde{\mathbf{f}}'(\tilde{\mathbf{u}}_0)\tilde{\mathbf{u}}_0 = \tilde{\mathbf{f}}'(\tilde{\mathbf{u}}_0)\tilde{\mathbf{u}} - \begin{bmatrix} 0 \\ 0 \\ f_{x\psi}|_{\tilde{\mathbf{u}}_0}\psi(t) \\ f_{y\psi}|_{\tilde{\mathbf{u}}_0}\psi(t) \\ \omega_z \\ 0 \\ 0 \\ f_{\phi\psi}|_{\tilde{\mathbf{u}}_0}\psi(t) \\ f_{\theta\psi}|_{\tilde{\mathbf{u}}_0}\psi(t) \\ f_{\psi\psi}|_{\tilde{\mathbf{u}}_0}\psi(t) \end{bmatrix},$$

such that

$$\tilde{\mathbf{f}}(\tilde{\mathbf{u}}) \approx \tilde{\mathbf{f}}(\tilde{\mathbf{u}}_0) + \tilde{\mathbf{f}}'(\tilde{\mathbf{u}}_0)(\tilde{\mathbf{u}} - \tilde{\mathbf{u}}_0) = \tilde{\mathbf{f}}'(\tilde{\mathbf{u}}_0)\tilde{\mathbf{u}} - \begin{bmatrix} 0 \\ 0 \\ f_{x\psi}\big|_{\tilde{\mathbf{u}}_0}\psi(t) \\ f_{y\psi}\big|_{\tilde{\mathbf{u}}_0}\psi(t) \\ 0 \\ 0 \\ 0 \\ f_{\phi\psi}\big|_{\tilde{\mathbf{u}}_0}\psi(t) \\ f_{\theta\psi}\big|_{\tilde{\mathbf{u}}_0}\psi(t) \\ f_{\psi\psi}\big|_{\tilde{\mathbf{u}}_0}\psi(t) \end{bmatrix}$$

$$= \begin{bmatrix} 0 & 0 & 1 & 0 & 0 & 0 & 0 & 0 & 0 & 0 \\ 0 & 0 & 0 & 1 & 0 & 0 & 0 & 0 & 0 & 0 \\ f_{xx}\big|_{\tilde{\mathbf{u}}_0} & f_{xy}\big|_{\tilde{\mathbf{u}}_0} & 0 & 0 & 0 & f_{x\theta}\big|_{\tilde{\mathbf{u}}_0} & f_{x\phi}\big|_{\tilde{\mathbf{u}}_0} & 0 & 0 & 0 \\ f_{yx}\big|_{\tilde{\mathbf{u}}_0} & f_{yy}\big|_{\tilde{\mathbf{u}}_0} & 0 & 0 & 0 & f_{y\theta}\big|_{\tilde{\mathbf{u}}_0} & f_{y\phi}\big|_{\tilde{\mathbf{u}}_0} & 0 & 0 & 0 \\ 0 & 0 & 0 & 0 & 0 & 0 & 0 & 0 & 0 & 1 \\ 0 & 0 & 0 & 0 & 0 & 0 & 0 & \sin(\psi(t)) & \cos(\psi(t)) & 0 \\ 0 & 0 & 0 & 0 & 0 & 0 & 0 & \cos(\psi(t)) & -\sin(\psi(t)) & 0 \\ f_{\phi x}\big|_{\tilde{\mathbf{u}}_0} & f_{\phi y}\big|_{\tilde{\mathbf{u}}_0} & 0 & 0 & 0 & f_{\phi\theta}\big|_{\tilde{\mathbf{u}}_0} & f_{\phi\phi}\big|_{\tilde{\mathbf{u}}_0} & 0 & -\omega_z & 0 \\ f_{\theta x}\big|_{\tilde{\mathbf{u}}_0} & f_{\theta y}\big|_{\tilde{\mathbf{u}}_0} & 0 & 0 & 0 & f_{\theta\theta}\big|_{\tilde{\mathbf{u}}_0} & f_{\theta\phi}\big|_{\tilde{\mathbf{u}}_0} & \omega_z & 0 & 0 \\ f_{\psi x}\big|_{\tilde{\mathbf{u}}_0} & f_{\psi y}\big|_{\tilde{\mathbf{u}}_0} & 0 & 0 & 0 & f_{\psi\theta}\big|_{\tilde{\mathbf{u}}_0} & f_{\psi\phi}\big|_{\tilde{\mathbf{u}}_0} & 0 & 0 & 0 \end{bmatrix}\tilde{\mathbf{u}}.$$

Noting that $f_{\psi x}\big|_{\tilde{\mathbf{u}}_0} = f_{\psi y}\big|_{\tilde{\mathbf{u}}_0} = 0$ and $f_{\psi\theta}\big|_{\tilde{\mathbf{u}}_0} = f_{\psi\phi}\big|_{\tilde{\mathbf{u}}_0} = 0$ due to the absence of a yaw-restoring mechanism, we get

$$\tilde{\mathbf{f}}(\tilde{\mathbf{u}}) \approx \begin{bmatrix} 0 & 0 & 1 & 0 & 0 & 0 & 0 & 0 & 0 & 0 \\ 0 & 0 & 0 & 1 & 0 & 0 & 0 & 0 & 0 & 0 \\ f_{xx}\big|_{\tilde{\mathbf{u}}_0} & f_{xy}\big|_{\tilde{\mathbf{u}}_0} & 0 & 0 & 0 & f_{x\theta}\big|_{\tilde{\mathbf{u}}_0} & f_{x\phi}\big|_{\tilde{\mathbf{u}}_0} & 0 & 0 & 0 \\ f_{yx}\big|_{\tilde{\mathbf{u}}_0} & f_{yy}\big|_{\tilde{\mathbf{u}}_0} & 0 & 0 & 0 & f_{y\theta}\big|_{\tilde{\mathbf{u}}_0} & f_{y\phi}\big|_{\tilde{\mathbf{u}}_0} & 0 & 0 & 0 \\ 0 & 0 & 0 & 0 & 0 & 0 & 0 & 0 & 0 & 1 \\ 0 & 0 & 0 & 0 & 0 & 0 & 0 & \sin(\psi(t)) & \cos(\psi(t)) & 0 \\ 0 & 0 & 0 & 0 & 0 & 0 & 0 & \cos(\psi(t)) & -\sin(\psi(t)) & 0 \\ f_{\phi x}\big|_{\tilde{\mathbf{u}}_0} & f_{\phi y}\big|_{\tilde{\mathbf{u}}_0} & 0 & 0 & 0 & f_{\phi\theta}\big|_{\tilde{\mathbf{u}}_0} & f_{\phi\phi}\big|_{\tilde{\mathbf{u}}_0} & 0 & -\omega_z & 0 \\ f_{\theta x}\big|_{\tilde{\mathbf{u}}_0} & f_{\theta y}\big|_{\tilde{\mathbf{u}}_0} & 0 & 0 & 0 & f_{\theta\theta}\big|_{\tilde{\mathbf{u}}_0} & f_{\theta\phi}\big|_{\tilde{\mathbf{u}}_0} & \omega_z & 0 & 0 \\ 0 & 0 & 0 & 0 & 0 & 0 & 0 & 0 & 0 & 0 \end{bmatrix} \begin{bmatrix} x \\ y \\ v_x \\ v_y \\ \psi(t) \\ \theta \\ \phi \\ \omega_x \\ \omega_y \\ \omega_z \end{bmatrix}.$$

From this, it follows that

$$\dot{\omega}_z(t) \approx 0 \rightarrow \omega_z = \text{constant},$$

$$\dot{\psi}(t) \approx \omega_z(t) = \omega_z \rightarrow \psi(t) = \omega_z t,$$

and we can reduce the ten-dimensional vectorial differential equation by two more dimensions to

$$\hat{\mathbf{f}}(\hat{\mathbf{u}}) \approx \hat{\mathbf{J}}(\hat{\mathbf{u}}_0, t)\hat{\mathbf{u}},$$

with

$$\hat{\mathbf{J}}(\hat{\mathbf{u}}_0,t)\hat{\mathbf{u}} = \begin{bmatrix} 0 & 0 & 1 & 0 & 0 & 0 & 0 & 0 \\ 0 & 0 & 0 & 1 & 0 & 0 & 0 & 0 \\ f_{xx}|_{\hat{\mathbf{u}}_0} & f_{xy}|_{\hat{\mathbf{u}}_0} & 0 & 0 & f_{x\theta}|_{\hat{\mathbf{u}}_0} & f_{x\phi}|_{\hat{\mathbf{u}}_0} & 0 & 0 \\ f_{yx}|_{\hat{\mathbf{u}}_0} & f_{yy}|_{\hat{\mathbf{u}}_0} & 0 & 0 & f_{y\theta}|_{\hat{\mathbf{u}}_0} & f_{y\phi}|_{\hat{\mathbf{u}}_0} & 0 & 0 \\ 0 & 0 & 0 & 0 & 0 & 0 & \sin(\omega_z t) & \cos(\omega_z t) \\ 0 & 0 & 0 & 0 & 0 & 0 & \cos(\omega_z t) & -\sin(\omega_z t) \\ f_{\phi x}|_{\hat{\mathbf{u}}_0} & f_{\phi y}|_{\hat{\mathbf{u}}_0} & 0 & 0 & f_{\phi\theta}|_{\hat{\mathbf{u}}_0} & f_{\phi\phi}|_{\hat{\mathbf{u}}_0} & 0 & -\omega_z \\ f_{\theta x}|_{\hat{\mathbf{u}}_0} & f_{\theta y}|_{\hat{\mathbf{u}}_0} & 0 & 0 & f_{\theta\theta}|_{\hat{\mathbf{u}}_0} & f_{\theta\phi}|_{\hat{\mathbf{u}}_0} & \omega_z & 0 \end{bmatrix} \begin{bmatrix} x \\ y \\ v_x \\ v_y \\ \theta \\ \phi \\ \omega_x \\ \omega_y \end{bmatrix},$$

and

$$\hat{\mathbf{u}}_0 = \mathbf{0}.$$

To continue with the stability analysis, given that we now have a linear *time-dependent* system for spinning lightsails, we need to find the monodromy matrix, i.e., the state transition matrix after one full period $T = 2\pi/\omega_z$. To find the general state transition matrix, we need to solve for

$$\dot{\mathbf{\Phi}}(t,0) = \hat{\mathbf{J}}(\hat{\mathbf{u}}_0,t)\mathbf{\Phi}(t,0); \qquad \mathbf{\Phi}(0,0) = \mathbf{I}.$$

$\mathbf{\Phi}(t = T, 0)$ can be found via numerical integration of the differential equation. Specifically, writing the state transition matrix $\mathbf{\Phi}(t,0)$ in terms of its row vectors

$$\mathbf{\Phi}(t,0) = \begin{bmatrix} \mathbf{a}_1^T \\ \mathbf{a}_2^T \\ \vdots \\ \mathbf{a}_7^T \\ \mathbf{a}_8^T \end{bmatrix},$$

with $\mathbf{a}_i = \{a_{i1}, a_{i2}, \dots, a_{i8}\}^T \in \mathbb{R}^8, i = 1, \dots, 8$ being an eight-dimensional column vector, we can express the differential equation above in vectorial form as

$$\begin{bmatrix} \dot{\mathbf{a}}_1 \\ \dot{\mathbf{a}}_2 \\ \vdots \\ \dot{\mathbf{a}}_7 \\ \dot{\mathbf{a}}_8 \end{bmatrix} = \begin{bmatrix} \left(\mathbf{j}_1^T \cdot \mathbf{\Phi}(t,0)\right)^T \\ \left(\mathbf{j}_2^T \cdot \mathbf{\Phi}(t,0)\right)^T \\ \vdots \\ \left(\mathbf{j}_7^T \cdot \mathbf{\Phi}(t,0)\right)^T \\ \left(\mathbf{j}_8^T \cdot \mathbf{\Phi}(t,0)\right)^T \end{bmatrix},$$

with the 64-dimensional state vector $\mathbf{s} = [a_{11}(t), \dots, a_{18}(t), a_{21}(t), \dots, a_{28}(t), \dots, a_{88}(t)]^T$.

We solved this vector differential equation of first order in MATLAB using ode45 to obtain a numerical result for $\mathbf{\Phi}(t = T, 0)$. Finally, using MATLAB's eigenvalue solver, we calculated the eight eigenvalues of $\mathbf{\Phi}(t = T, 0)$ to be

$$\lambda_{1,2} \approx 0.9859 \pm 0.1671i,$$

$$\lambda_{3,4} \approx 0.9906 \pm 0.1367i,$$

$$\lambda_{5,6} \approx 0.9995 \pm 0.0308i,$$

$$\lambda_{7,8} \approx 0.9999 \pm 0.0001i.$$

The absolute values of the complex eigenvalues, $|\lambda_i|$ for $i = 1, \dots, 8$, are then given by

$$|\lambda_i| \approx 1\ \forall i,$$

rounded off to four decimal places.

According to Floquet theory, with the absolute values of the eight eigenvalues being on the unit circle in the complex plane, we can conclude that the presented composite metagrating design enables marginal stability in linearized dynamics of spinning flat lightsails. However, to verify that actual trajectories with finite initial conditions are bounded, the corresponding equations of motion need to be numerically evolved.

Supplementary Note 7: Calculating optical forces for metagratings

Prior to simulated release and propulsion of the mesh-based lightsail, each triangular mesh element m is characterized by a normal vector $\mathbf{n}_m$ and a "grating" vector $\mathbf{g}_m$ being parallel to the metagratings this particular mesh element is meant to host. After launch, both vectors will evolve according to the specific rotation of that mesh element, now described by a *rotated* normal vector $\tilde{\mathbf{n}}_m$ and a *rotated* grating vector $\tilde{\mathbf{g}}_m$ at time t. Assuming all vectors to be normalized to a length of 1, we can then calculate the matrix that describes rotation of mesh element m as

$$\mathbf{R}_m = \begin{bmatrix} \tilde{g}_{x,m} & \tilde{n}_{x,m} & (\tilde{\mathbf{g}}_m \times \tilde{\mathbf{n}}_m)_x \\ \tilde{g}_{y,m} & \tilde{n}_{y,m} & (\tilde{\mathbf{g}}_m \times \tilde{\mathbf{n}}_m)_y \\ \tilde{g}_{z,m} & \tilde{n}_{z,m} & (\tilde{\mathbf{g}}_m \times \tilde{\mathbf{n}}_m)_z \end{bmatrix} \begin{bmatrix} g_{x,m} & g_{y,m} & g_{z,m} \\ n_{x,m} & n_{y,m} & n_{z,m} \\ (\mathbf{g}_m \times \mathbf{n}_m)_x & (\mathbf{g}_m \times \mathbf{n}_m)_y & (\mathbf{g}_m \times \mathbf{n}_m)_z \end{bmatrix}.$$

Knowledge of the components of $\mathbf{R}_m = \mathbf{H}_B^I(\psi_m, \theta_m, \phi_m)$ allows us to calculate the Euler angles of respective mesh element

$$\theta_m = \sin^{-1}(R_{13,m}),$$

$$\phi_m = -\tan^{-1}\left(\frac{R_{23,m}}{R_{33,m}}\right),$$

$$\psi_m = -\tan^{-1}\left(\frac{R_{12,m}}{R_{11,m}}\right),$$

where we use MATLAB's four-quadrant inverse tangent function atan2 in our code for correct calculation of ϕ_m and ψ_m.

After looking up the tabulated pressure $\mathbf{p}_m$ on mesh element m based on its calculated Euler angles (θ_m, ϕ_m) (noting that our pressures do not depend on ψ due to the assumption of a synchronously spinning electric field), we can determine the optically induced force on the specific mesh element in the body frame to be

$$\mathbf{F}_m^B = \cos(\theta_m)\cos(\phi_m)\, A_m \mathbf{p}_m(\theta_m, \phi_m) I_m,$$

where A_m is the area of the mesh element and I_m is the discretized value of the Gaussian intensity function present on the mesh element. As before, optical forces in the inertial frame are obtained by multiplication of $\mathbf{F}_m^B$ with the direction cosine matrix as follows:

$$\mathbf{F}_m = \mathbf{H}_B^I(\psi_m, \theta_m, \phi_m)\mathbf{F}_m^B.$$

Due to the interconnected nature of mesh elements, torque contributions are inherently accounted for by the collective effect of optical forces on individual mesh elements.

Supplementary Note 8: Alternative design for yaw-restoring torque

All self-stabilizing dynamics of flat spinning flexible lightsails patterned with optical metagratings assume perfect yaw-angle alignment between the electric field of the incident laser beam and the orientation of the metagratings. This synchronous rotation of the polarization with the spinning lightsail allowed us to neglect any dependence of the optical pressures on the yaw angle ψ, obtain results of sufficient acceleration duration with reasonable computation time, and perform Floquet analysis. Consequently, our claim of self-stabilization is based on self-restoring in-plane forces and self-stabilizing torques about the x- and y-axis, but not the z-axis.

Robustness to perturbations to the alignment between the angular velocity of the laser beam and the angular velocity of the lightsail could be introduced with an altered design, in which the metagrating sections are rotated with respect to each[36]. One potential design is shown in Supplementary Fig. 9a, with the TE metagratings (colored in blue) being rotated by $\delta = 5°$ relative to the lightsail's y-axis y_{BF}. Such a modification barely affects the original shape and magnitude of the angle-dependent forces $F_x(\theta)$ and $F_y(\phi)$ (Supplementary Fig. 9b). The same observation of minor changes to the self-restoring torques $\tau_x(\phi)$ and $\tau_y(\theta)$ can also be made (Supplementary Fig. 9c), suggesting that the self-stabilization mechanism will be preserved. Importantly, a self-restoring torque about the z-axis emerges in this modified design, allowing to potentially realign the angular velocity of the lightsail to the beam's angular velocity upon perturbations about the z-axis.

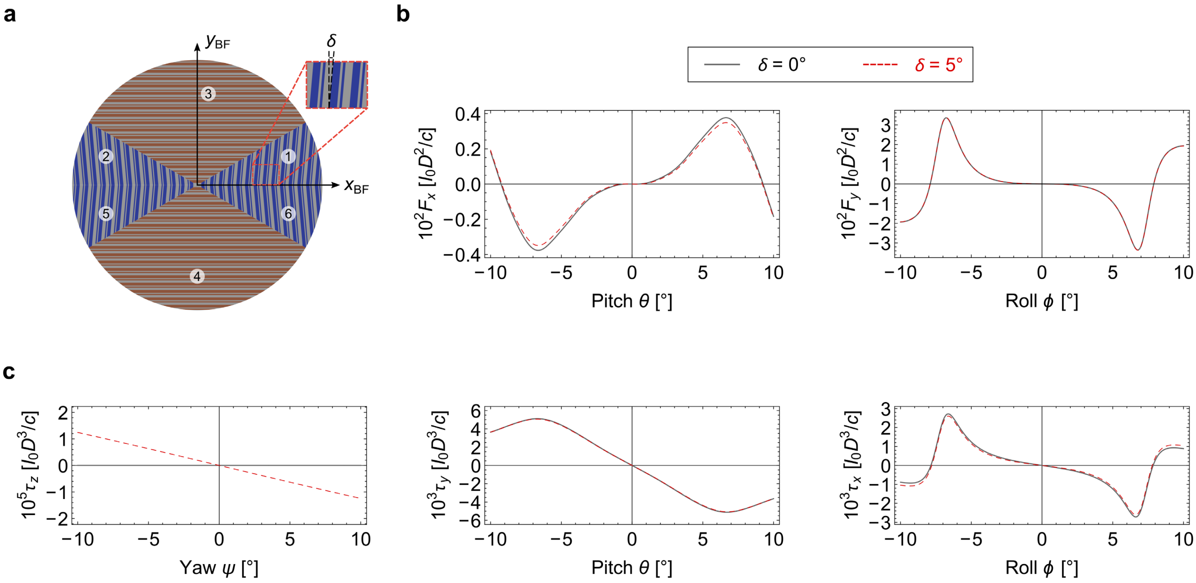

Supplementary Figure 9. Composite metagrating design for self-restoring torque about z-axis. (a) Original design is modified through rotation of TE metagratings by angle δ relative to the lightsail's y-axis y_{BF}. **(b)** Force pairs $F_x(\theta)$ and $F_y(\phi)$ induced in original (solid lines) and modified (dashed lines) design, displaying very similar behavior. **(c)** Torque $\tau_z(\psi)$ emerging in modified design exhibits correct polarity for introducing robustness to misalignment between the angular velocities of the lightsail and laser beam, while torque pairs $\tau_y(\theta)$ and $\tau_x(\phi)$ maintain their restoring shape.

In contrast to the original design shown in Fig. 5, the modified design comprises six distinct regions, for each of which the electric field vector is transformed according to

$$\mathbf{E}_i = \mathbf{H}_I^B(\psi + \beta_j, \theta, \phi)\mathbf{E}_I,$$

with $\boldsymbol{E}_I = E_0(0, 1, 0)^T$ and $\beta_{1,2,\ldots,6} = -\delta, \pi + \delta, \pi/2, 3\pi/2, \pi - \delta, \delta$.

Similarly, the wavevector for each of the six regions can be calculated as

$$\mathbf{k}_i = \mathbf{H}_I^B(\psi + \beta_j, \theta, \phi)\mathbf{k}_I.$$

In contrast to the case of $\psi = \delta = 0$, optical pressures $\mathbf{p}_i(\psi, \theta, \phi)$ in the local frame will now need to be simulated for all six regions, from which the optical pressures in the body frame, and consequently the forces and torques shown in Supplementary Fig. 9 can be calculated following our description in Supplementary Note 3 above.

Supplementary Note 9: Example passively stabilized metagrating sail w/ initial translation

To further probe the stability of the reported metagrating-based lightsail design, we simulated another case where the lightsail is initially displaced, but not tilted. Specifically, we assumed an initial translation offset of $x_0 = y_0 = 0.05$ m as in the case discussed in the main text. Setting the initial tilt offset to zero leads to considerably smaller tilt deviations and lateral displacements throughout the flight, as is evident from the snapshots shown in Supplementary Fig. 10a. An animation of this simulation is available as Supplementary Video 4. The temperature distribution and peak temperature is generally similar to that of Fig. 6a. In terms of the trajectory and translational degrees of freedom, both the rigid and the flexible lightsails appear to exhibit passive stability as evidenced by their similar bounded transverse motion; in fact, the trajectories are more regular and less complex than those shown in Fig. 6. Lateral displacements of up to 0.08 m are observed for both the rigid and flexible case (Supplementary Fig. 10b and Supplementary Fig. 10c), with more apparent, yet small deviations starting to appear after the first second of acceleration.

As for the first case discussed in the main text, we observe multiple frequency components within the simulated trajectories and tilt angles (Supplementary Fig. 10d–g), with the most noticeable one being again the slow frequency component at 240 Hz superimposed upon slower frequencies of approximately 2.5 Hz and 0.6 Hz. The observation of displacement along x and y being more tightly confined can also be made for the pitch and roll angles of the rigid lightsail, as they remain bounded within ±1.3° during the simulated time span, suggesting a lesser degree of deformation and vibration in the membrane. The temporal evolution of pitch and roll angle distributions of the flexible lightsail again follows closely θ and ϕ of the rigid lightsail, confirming that spin stabilization at 120 Hz is sufficiently fast to treat our flexible lightsail as quasi-rigid. Nevertheless, we note that a finite angular spread of pitch and roll angles of ~1° can be observed for all mesh elements constituting the flexible lightsail. Finally, due to the discretized surface of the flexible lightsail, signs of mesh elements on the perimeter experiencing larger rotations remain visible in the insets of Supplementary Fig. 10fg despite truncating histogram bins with only few elements (less than 10 within bins of width 0.05°).

We note that in contrast to the result shown in Fig. 6, the dynamics of the flat flexible lightsail with initial translation only was simulated via symplectic Euler integration.

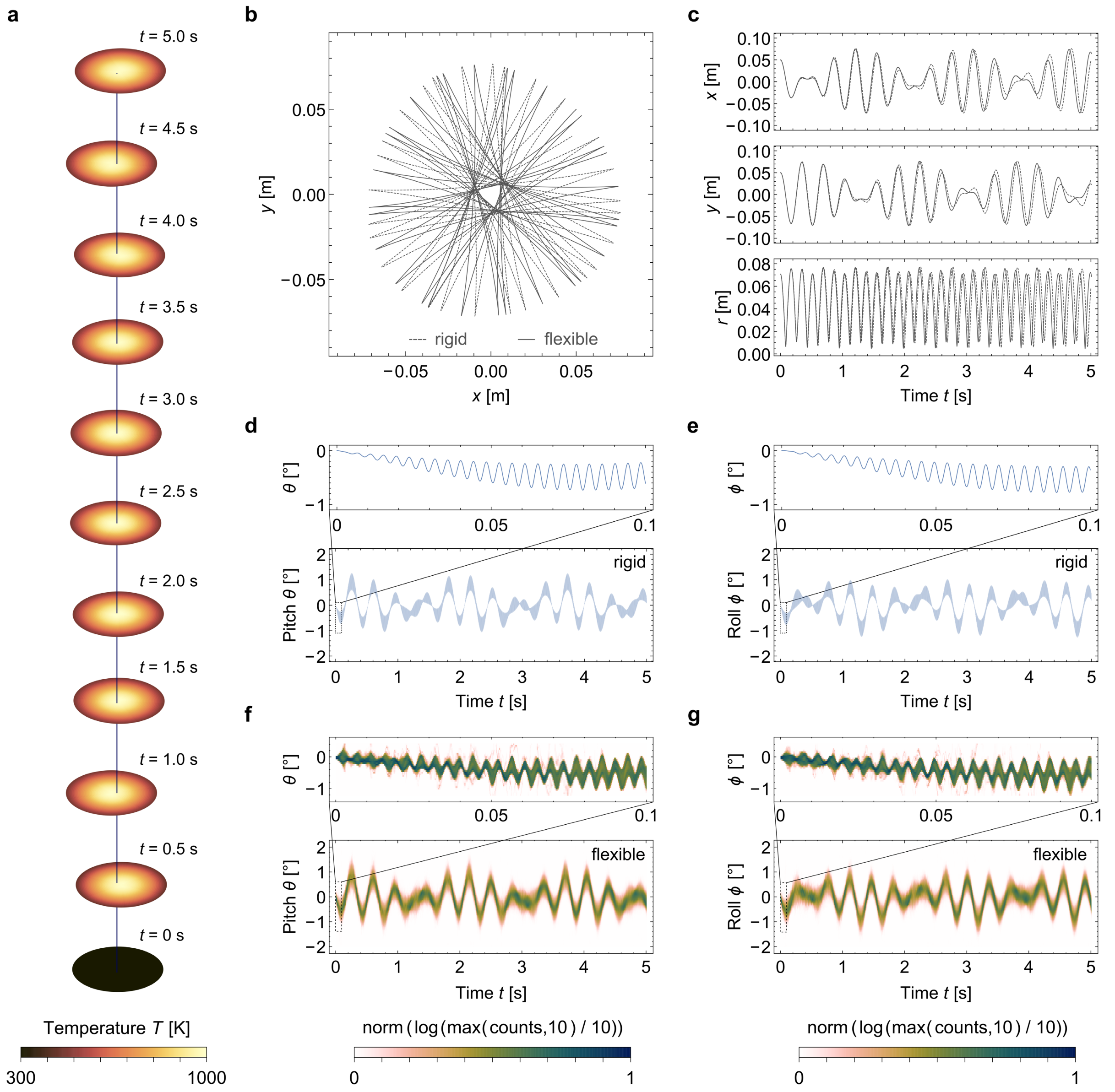

Supplementary Figure 10. Acceleration dynamics of a flexible and a rigid spinning composite metagrating lightsail subject to an initial translational offset ($D = 1$ m, $t_{\text{eff}}^{\text{TE}} = 400$ nm and $t_{\text{eff}}^{\text{TM}} = 413.3$ nm). Lightsails are initially offset by $x_0 = y_0 = 0.05D = 50$ mm relative to the beam center. **(a)** Snapshots of the beam-riding flexible lightsail's position, angular orientation, temperature, and shape at different times. **(b)** Lightsail trajectory throughout the 5 s simulation duration. (**c)** Lightsail x- and y-position and radial distance r from the beam center versus time for the flexible and rigid version of the same lightsail, exhibiting bounded and oscillation around the equilibrium at $x, y = 0$. **(d), (e)** Evolution of pitch θ and roll ϕ, respectively, of the rigid lightsail versus time, showing multi-frequency oscillation around the equilibrium at θ, $\phi = 0$. **(f), (g)** Distribution of θ and ϕ angles, respectively, of all mesh elements comprising the flexible lightsail versus time, with the color bar depicting normalized counts on a logarithmic scale with a bin width of 0.05°, showing both bounded oscillations and limited angular spread, although minor shape distortion is observed via the range of surface tilt angles at any given time. For **(d) – (g)**, insets show fast-frequency oscillations within a reduced time window (0.1 s).

Supplementary Note 10: Deviation from flatness for flexible spinning lightsails

To study the shape evolution of the studied flexible metagrating-based lightsail, it is helpful to compare its current state of deformation to a reference rigid lightsail. Such a flat shape can be defined at any time step t_i by the center of mass coordinates $\mathbf{r}_{\text{CoM}} = (x_{\text{CoM}}, y_{\text{CoM}}, z_{\text{CoM}})$ and averaged pitch $\bar{\theta}$, roll $\bar{\phi}$ and yaw $\bar{\psi}$ angles, i.e., $\bar{\alpha} = \sum_m \alpha_m / m$, where $\alpha_m \in \{\theta_m, \phi_m, \psi_m\}$ for the corresponding Euler angle of mesh element m. During the entire simulation duration, the node coordinates $\mathbf{r}_n$ and centroids $\mathbf{c}_m$ of every mesh element is being tracked, thus allowing to calculate the displacement from a flat shape as

$$d_{\text{node}} = \frac{|(\mathbf{r}_{\text{CoM}} - \mathbf{r}_n) \cdot \mathbf{n}_{\text{flat}}|}{|\mathbf{n}_{\text{flat}}|}, \qquad d_{\text{centroid}} = \frac{|(\mathbf{r}_{\text{CoM}} - \mathbf{c}_m) \cdot \mathbf{n}_{\text{flat}}|}{|\mathbf{n}_{\text{flat}}|},$$

and to show the distribution of node or centroid displacement within the flexible lightsail from a flat shape in Supplementary Fig. 11, or the maximum displacement in Supplementary Fig. 12.

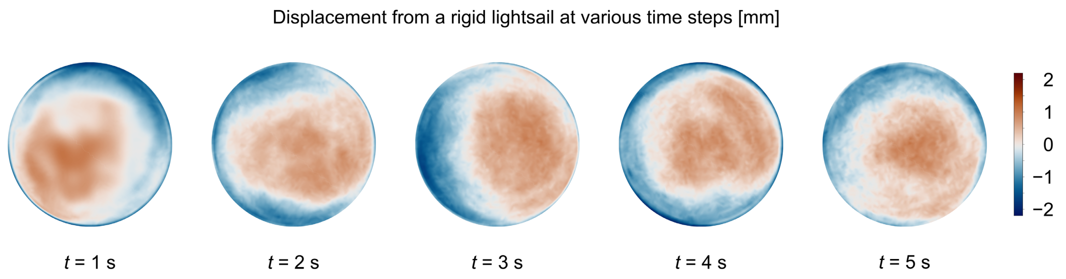

Supplementary Figure 11. Deviation from flatness of flexible spinning lightsail (initial translation of $x_0 = y_0 = 0.05$ m and initial tilt of $\theta_0 = \phi_0 = -2°$) simulated in Fig. 6 from a flat (rigid) lightsail (shape) at various time steps.

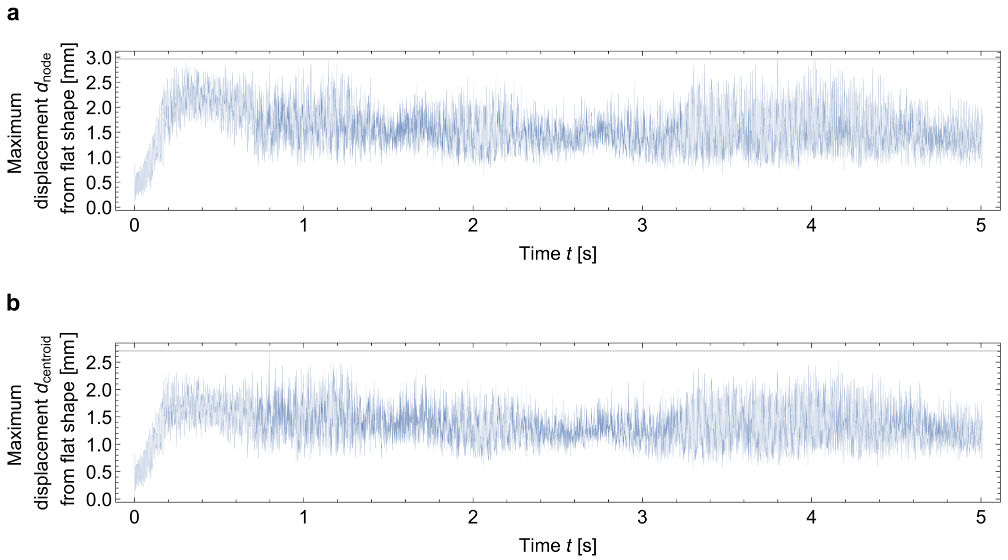

Supplementary Figure 12. Maximum displacement of the flexible spinning lightsail (initial translation of $x_0 = y_0 = 0.05$ m and initial tilt of $\theta_0 = \phi_0 = -2°$) simulated in Fig. 6 from a flat (rigid) shape versus time in terms of its **(a)** mesh nodes and **(b)** centroids of mesh elements.

Supplementary Note 11: Temperature & strain analysis for stabilized metagrating lightsail

As mentioned in the main text, our flexible lightsail simulator stores several state variables of interest for post-processing and analysis, including the peak and average temperature of the lightsail during propulsion and the maximum strain on the lightsail due to mechanical forces and thermal expansion, downsampled by a factor of 8 for memory management. Due to the underfilling beam width of $w = 0.4D$, regardless of whether the lightsail is initially only translated or also tilted, the difference between the peak, average and minimum temperature of points on the lightsail can be several hundreds of Kelvin (Supplementary Fig. 13). While the center of the lightsail heats up to a peak temperature of just below 1000 K during propulsion, its perimeter or edge points experience a temperature rise of less than 200 K, the difference of which results in an average temperature in between these two extremes. Including an initial tilt to the simulated trajectories induces more variation in especially both peak (center) and minimum (perimeter) temperatures of the accelerated lightsail. While the simulated maximum temperature appears sufficiently below silicon nitride's theoretical vacuum decomposition temperature, we note that it is difficult to estimate the practical limiting temperature for Si_3N_4 lightsails based on properties reported in literature, owing to the diversity of applications, the varying stoichiometry, density, and stress produced by CVD methods, and the relative complexity of the N-Si system at high temperatures. More experimental efforts will be needed to probe this practical limit for Si_3N_4 lightsails.

While the maximum temperature is of interest for assessing the need to include temperature-dependent material properties and requirements for payload integration, insights on the maximum strain help to deduce how close the lightsail is to mechanical failure and breaking apart. As seen in Supplementary Fig. 14, for both studied cases discussed in the main text, the maximum strain stays below 0.001, which multiplied by stoichiometric silicon nitride's Young's modulus of 270 GPa is one order of magnitude lower than its tensile strength. We note that due to the effects of mesh discretization and chosen resolution given available computational resources, reported peak strain and thus stress values are being underestimated by ~10%.

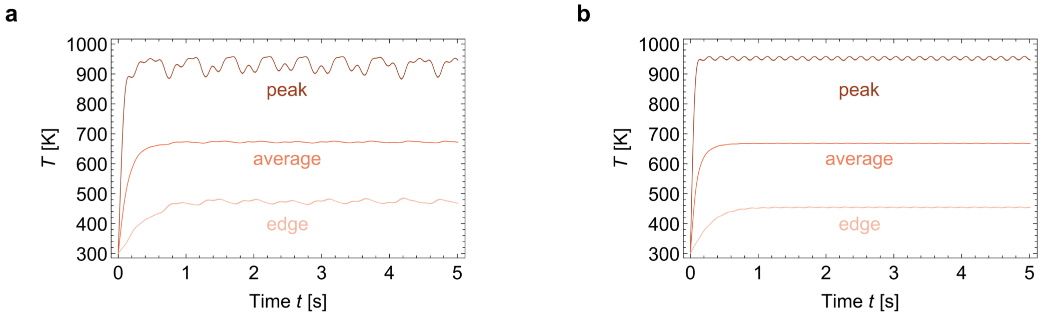

Supplementary Figure 13. Peak (brown) and average (orange) lightsail temperature versus time for cases discussed in the main text, where a flexible metagrating-patterned lightsail is either **(a)** being initially translated by $x_0 = y_0 = 0.05$ m and initially tilted by $\theta_0 = \phi_0 = -2°$, or **(b)** only initially translated by $x_0 = y_0 = 0.05$ m. Greater temperature variations are observed in (b) because the lightsail's lateral oscillations are considerably larger.

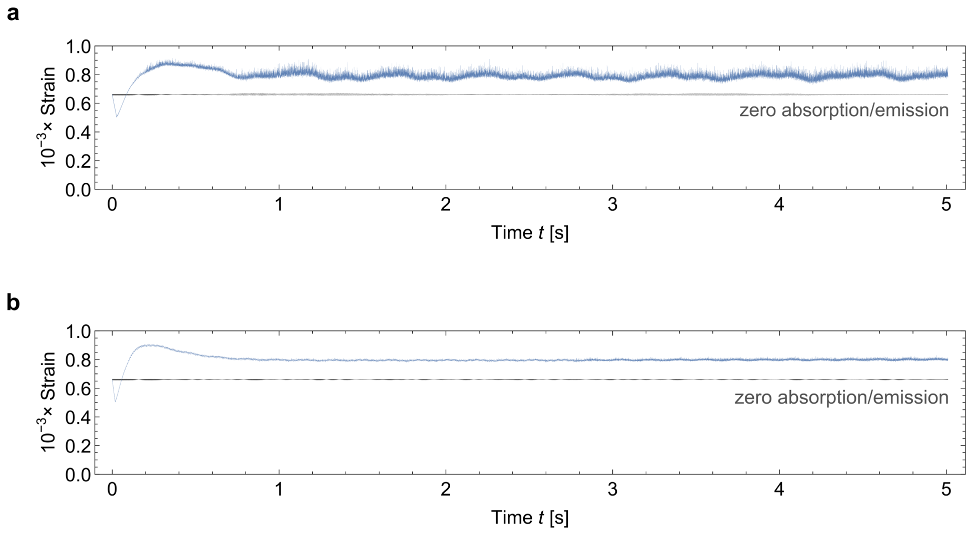

Supplementary Figure 14. Maximum strain on the flexible metagrating-patterned lightsails versus time for cases discussed in the main text, where a flexible, metagrating-patterned lightsail is either **(a)** being initially translated by $x_0 = y_0 = 0.05$ m and tilted by $\theta_0 = \phi_0 = -2°$, or **(b)** only initially translated by $x_0 = y_0 = 0.05$ m. Note that based on the modulus and tensile strength of Si_3N_4 (see Supplementary Table 1), the material strain limit is ~4%; over 40x greater than the peak simulated strain.

Supplementary Note 12: Effect of temperature on dynamics of flexible lightsails

To study the influence of finite absorption and thus temperature rise of flexible lightsails during propulsion on their dynamics, additional simulations were run with the absorptivity set to zero. This prevents the lightsail before heating up. At the same time, to avoid the lightsail from cooling down to unrealistically low temperatures, not taking into account the temperature of space, we also set the emissivity to zero. Results for the two case studies discussed in the main text are shown in Supplementary Fig. 15, comparing the trajectory of a flexible lightsail with finite absorptivity with that of a flexible lightsail with zero absorptivity and with that of a rigid lightsail with no heat transfer physics at all for an initial translation of $x_0 = y_0 = 0.05$ m and tilt $\theta_0 = \phi_0 = -2°$ (Supplementary Fig. 15a) and for initial translation $x_0 = y_0 = 0.05$ m only (Supplementary Fig. 15b). We observe that while all three cases share similar dynamical behavior, the trajectory of the non-absorbing flexible lightsail is distinct from the other two. This observation becomes clearer when looking at x and y versus time for all three cases in Supplementary Fig. 15c and Supplementary Fig. 15d. The slight deviation of the non-absorbing lightsail trajectory from the absorbing lightsail trajectory hints at the combined role of temperature *and* structural flexibility on the altered dynamics of a realistic, i.e., absorbing flexible lightsail when compared to its rigid counterpart.

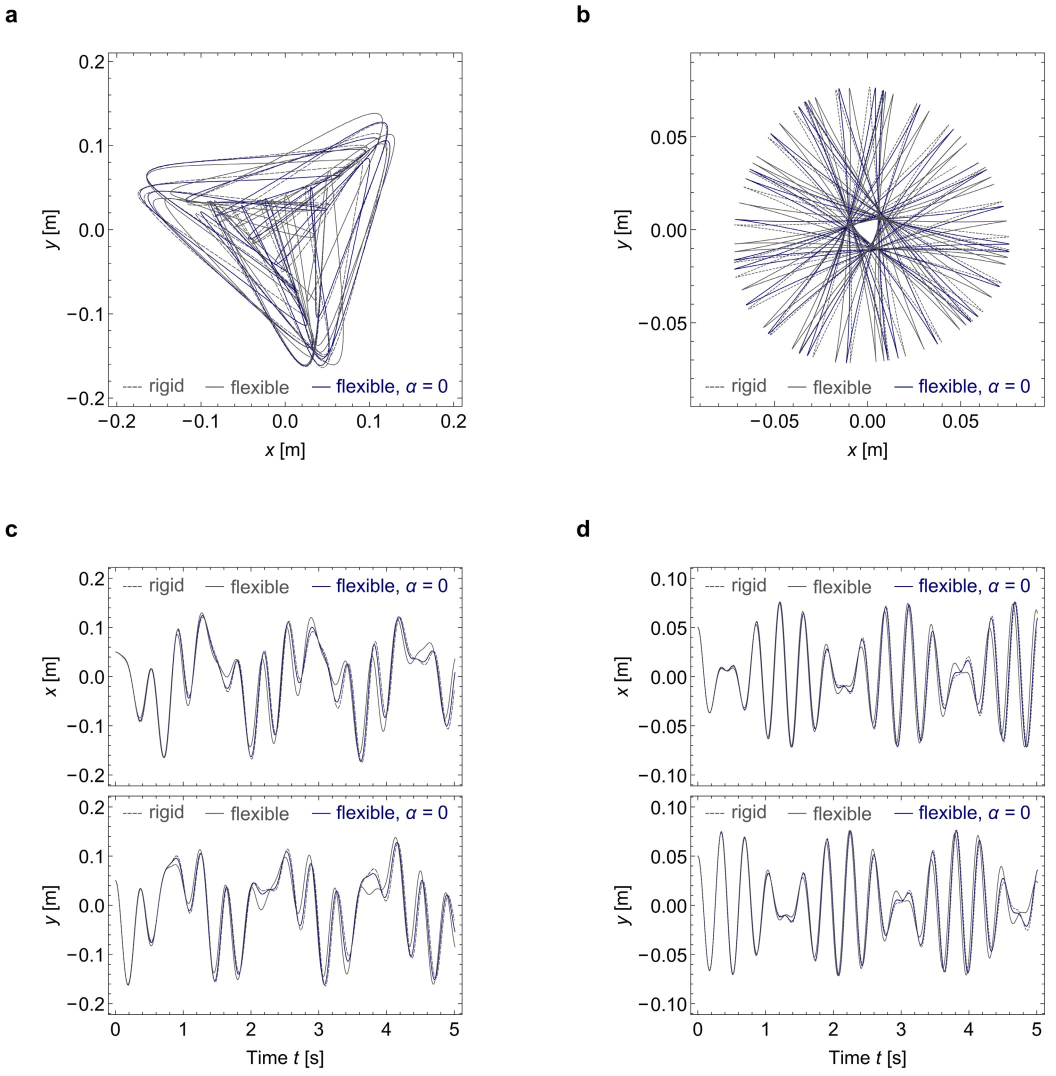

Supplementary Figure 15. Simulated x–y trajectories of rigid flat lightsail (gray dashed line), flexible flat lightsail (gray line) and flexible flat lightsail with zero absorption and emission (blue line), all patterned with the presented composite metagrating design for **(a)** initial translation of $x_0 = y_0 = 0.05$ m and initial tilt of $\theta_0 = \phi_0 = -2°$, and **(b)** initial translation of $x_0 = y_0 = 0.05$ m. The respective lightsail's x- and y-position are plotted versus time in **(c)** and **(d)** for both initial translation and rotation, and initial translation only, respectively, highlighting both similarities and differences and thus the combined role of thermal expansion and shape distortions on the flexible lightsail's dynamics.

Supplementary Note 13: Angular deviations of flexible lightsails from a flat shape

The angular deviations from a flat shape as illustrated in Supplementary Fig. 16 and Supplementary Fig. 17 can be calculated as $\theta_m - \bar{\theta}$ and $\phi_m - \bar{\phi}$, highlighting the presence of mechanical deformations of studied flexible lightsails despite spinning at high frequencies.

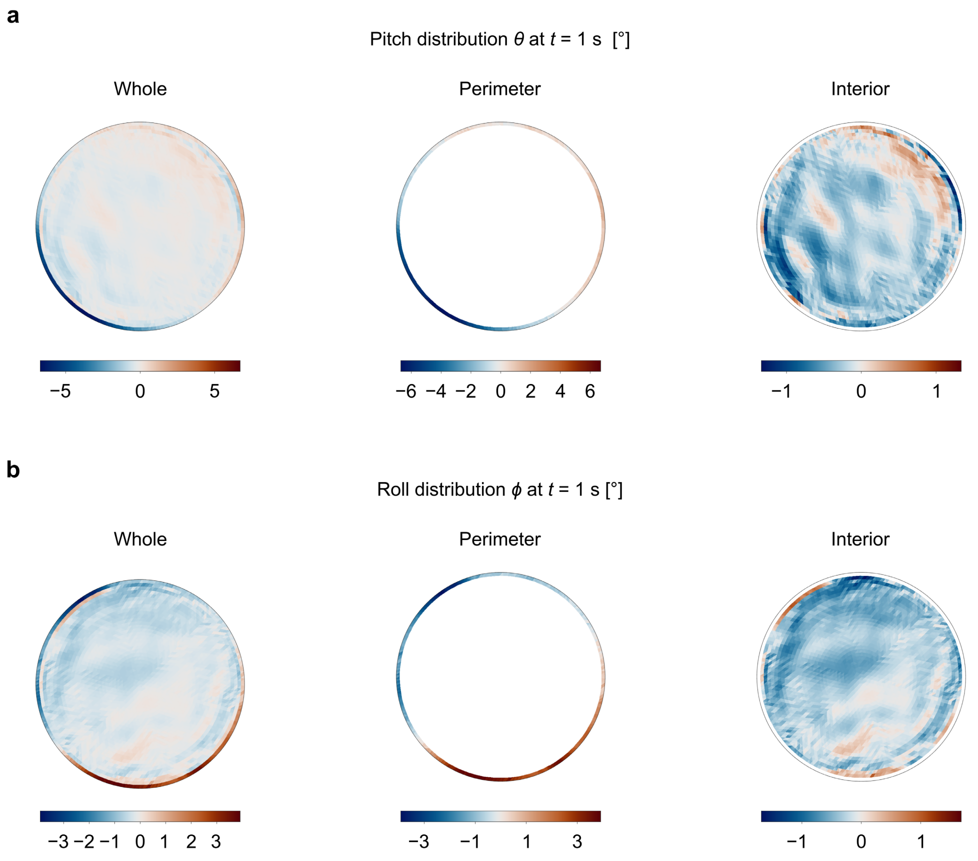

Supplementary Figure 16. (a) Pitch θ distribution and **(b)** roll ϕ distribution of mesh elements within the flexible spinning lightsail (initial translation of $x_0 = y_0 = 0.05$ m and initial tilt of $\theta_0 = \phi_0 = -2°$) simulated in Fig. 6 at time $t = 1$ s. Given the larger angles on the perimeter of the lightsail, the whole angle distribution (left) is shown separately in terms of its perimeter (middle) and interior (right).

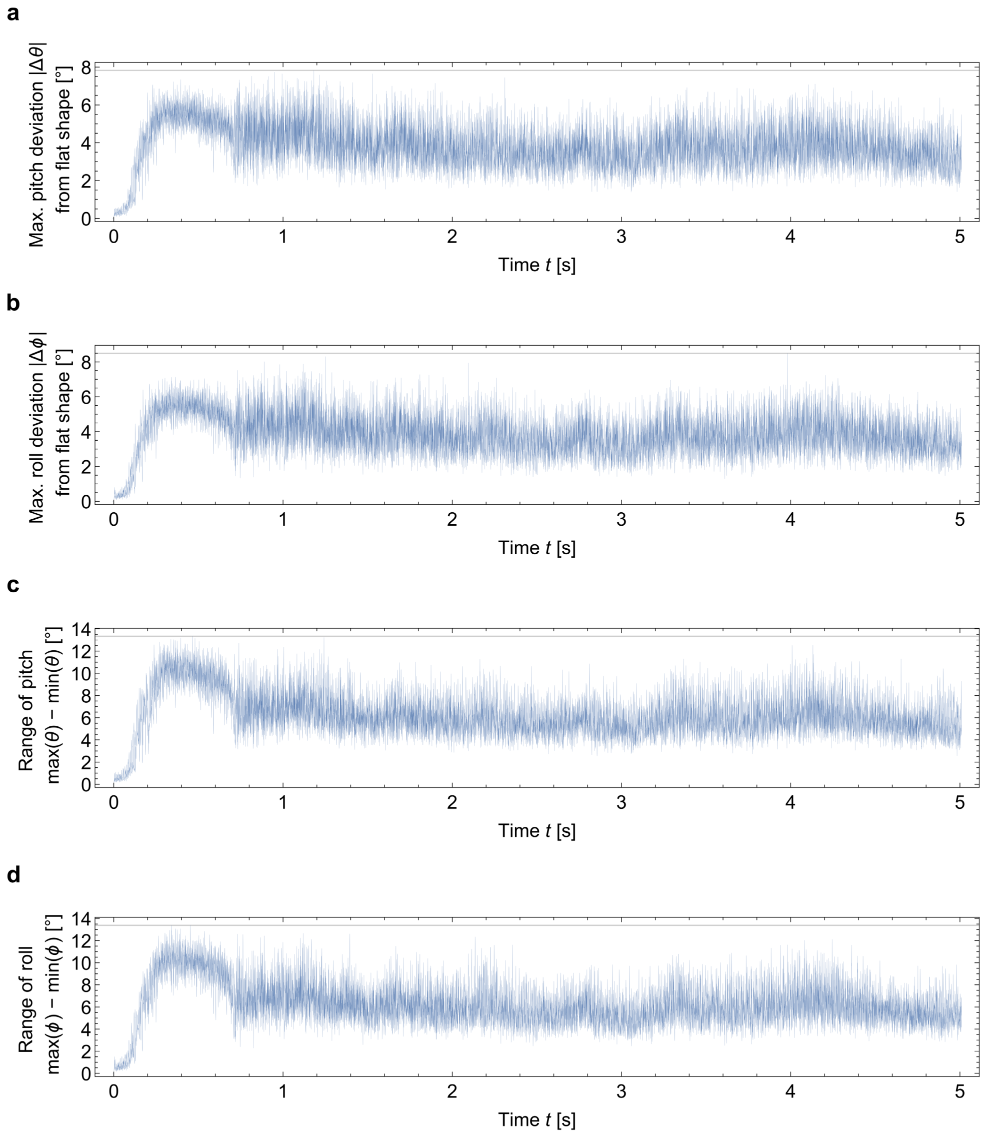

Supplementary Figure 17. Maximum angular deviations from a flat shape versus time for **(a)** pitch distribution θ and **(b)** roll distribution ϕ within the flexible spinning lightsail simulated in Fig. 6 (initial translation of $x_0 = y_0 = 0.05$ m and initial tilt of $\theta_0 = \phi_0 = -2°$). Range of **(c)** pitch angles θ and **(d)** roll angles ϕ within the flexible lightsail versus time.

Supplementary Note 14: Unstable cases of propelled flexible metagrating-based lightsails

The two cases discussed in the main text and in the previous section show passively stabilized dynamics of suitably designed metagrating-based lightsails with adequately high spinning frequency (120 Hz) and sufficiently small, i.e., underfilling propulsion beam width (0.4D). Changes to this parameter can result in unbounded and thus unstable trajectories assuming an initial translation of $x_0 = y_0 = 0.05$ m and initial tilt of $\theta_0 = \phi_0 = -2°$. For example, changing only the spinning frequency by reducing it from 120 Hz to 80 Hz results in unstable dynamics for the assumed initial conditions (Supplementary Fig. 18a). While Floquet theory still predicts marginal stability for our metagrating design at 80 Hz, it is the combination of a sufficiently large tilt and more structural deformation due to weaker spin-induced tensioning forces that explains the unstable behavior. The same observation of lost passive stabilization is made when increasing only the beam width instead by 25% to 0.5D (Supplementary Fig. 18b), possibly due to departure from the linear regime assumed in Floquet theory for the chosen finite initial tilt. Finally, the beam-riding stability depends on the restoring forces and torques produced by the chosen metagrating designs. It is likely that more optimal metagrating designs exist, but within our design space for the metagrating, a vast majority of design parameter choices produce unstable or less stable lightsails. One example is shown in Supplementary Fig. 18c: In this case, increasing the gap between resonators for both the TE and TM unit cells by 20% causes the lightsail to veer off from the beam path and become unstable within the first second of flight (Supplementary Fig. 18c). While all three cases were simulated to be unstable due to unbounded trajectories (Supplementary Fig. 18d), the third case of metagrating resonators being spaced farther apart can be explained theoretically. Increasing the distance flips the sign of the calculated torque τ_x,, which causes the lightsail to lose its roll-restoring ability and thus become unstable (Supplementary Fig. 18e). This conclusion is further corroborated by the fact that the complex eigenvalues of the corresponding monodromy matrix are calculated as

$$\lambda_{1,2} = 1.0666 \pm 0.043i,$$

$$\lambda_{3,4} = 0.9968 \pm 0.0805i,$$

$$\lambda_{5,6} = 0.9361 \pm 0.0377i,$$

$$\lambda_{7,8} = 0.9999 \pm 0.0001i,$$

rounded off to four decimal places, such that the absolute values of the complex eigenvalues, $|\lambda_i|$ for $i = 1, \dots, 8$, are then given by

$$|\lambda_{1,2}| \approx 1.0674, |\lambda_{3,4}| \approx 1, |\lambda_{5,6}| \approx 0.9368, |\lambda_{7,8}| \approx 0.9999.$$

In contrast to the original metagrating design, we see that the absolute values of the eigenvalues do not exclusively lie on the unit circle, indicating an unstable linear time-periodic system, or spinning lightsails that would not be passively (marginally) stabilized.

We note that absolute values of complex eigenvalues for cases A ($f = 80$ Hz) and B ($w = 0.5D$), are ~1 despite the simulated trajectories being unbounded, indicating that chosen initial conditions lie outside of the linear regime assumed by Floquet theory.

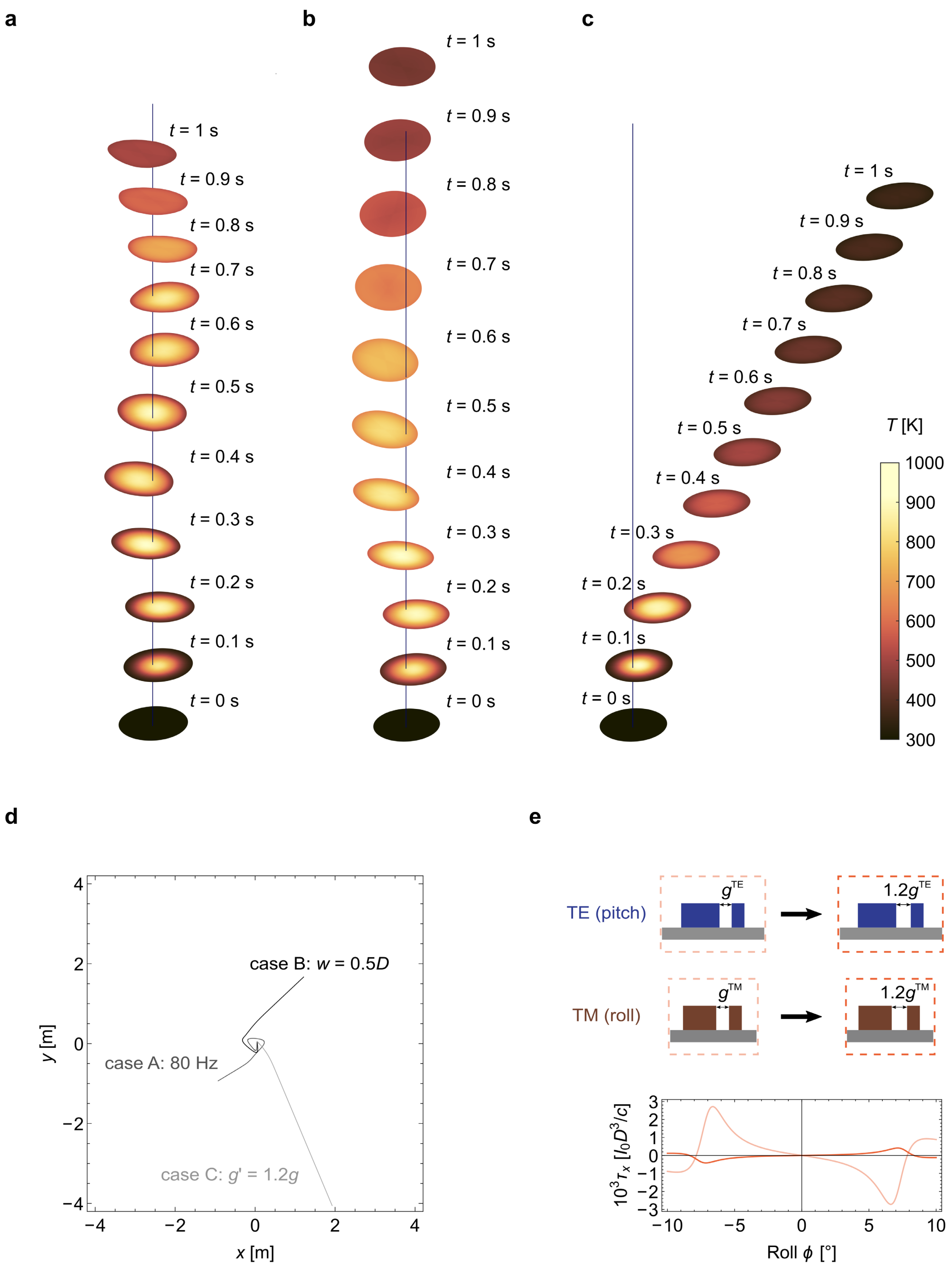

Supplementary Figure 18. Unstable propulsion of flexible, metagrating-patterned lightsails being initially translated by $x_0 = y_0 = 0.05$ m and tilted by $\theta_0 = \phi_0 = -2°$ due to **(a)** insufficient spin speed $f = 80$ Hz, **(b)** a larger beam width $w = 0.5D$, and **(c)** altered TE and TM metagrating unit cell designs, where the distance between resonators was increased by 20% compared to the stable designs presented in the main text. **(d)** Instability is characterized by the flexible lightsail veering off from the beam center for all three considered cases. **(e)** Specifically, increasing the distance between resonators results in the torque about x changing the sign of its slope and thus losing its roll-restoring ability.

Supplementary Note 15: Flexible vs. rigid metagrating sails at lower spin frequencies

While the self-stabilizing dynamics of flexible metagrating-based lightsails resembles corresponding rigid-body dynamics at high spin frequencies, when lowering the spin frequency, the amount of mechanical flexibility will increase, thus reducing the similarity between flexible and rigid lightsail trajectories. In Supplementary Fig. 19, we compare the acceleration dynamics of a flexible lightsail based on our composite metagrating design with its rigid version, following an initial misalignment of $x_0 = y_0 = 1$ cm and $\theta_0 = \phi_0 = -1°$, by simulating three distinct spin frequencies. For a spin frequency of 75 Hz, after two seconds of acceleration, both lightsails remain on a bounded trajectory. However, they travel visibly different paths in the *xy*-plane in comparison to the case of 120 Hz presented in Fig. 6. At a spin frequency of 60 Hz, even though both lightsails eventually escape the laser beam, they do so at different times. Specifically, the rigid lightsail spirals out of the laser beam immediately, reaching the arbitrarily chosen circumference at $r = 0.2$ m after 0.449 s. On the other hand, the flexible lightsail manages to stay in vicinity of the beam more than twice as long, encircling the beam center more than once before veering off-course and reaching $r = 0.2$ m at $t = 1.179$ s. Finally, if both lightsails are spinning at 71 Hz, the rigid lightsail flies away, while its flexible counterpart traverses a bounded trajectory, thus exhibiting self-stabilizing behavior throughout the entire simulated 2 s. This observation does not only highlight the importance and capabilities of our flexible lightsail simulator, but also suggests that structural flexibility in spinning lightsails could benefit self-stabilizing dynamics in certain cases. Longer simulations and more studies with many distinct initial conditions as well as a closer look at the role of mechanical deformations and modes will be needed to draw a definite conclusion.

The relative tolerance of the ode45 solver for simulating the rigid lightsails was chosen to be 10^{-8}.

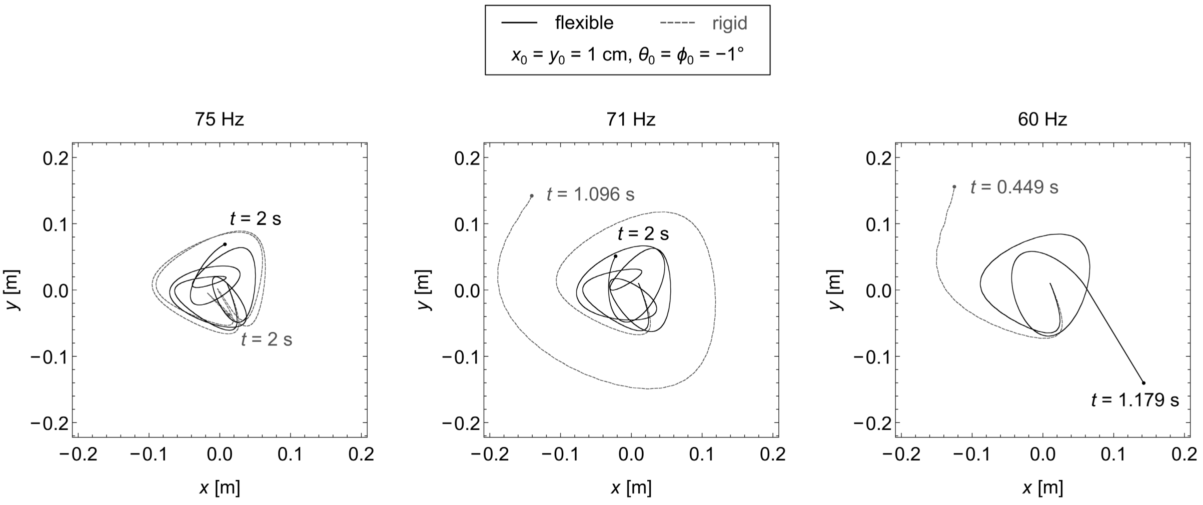

Supplementary Figure 19. Comparison of flexible and rigid lightsail dynamics at lower spin frequencies. At 75 Hz (left), both flexible and rigid lightsail remain on a bounded, yet distinctively different trajectory after the simulated duration of 2 s. Reducing the spin frequency to 71 Hz results in unstable rigid-body dynamics, while the flexible lightsail manages to stay in vicinity of the beam center, suggesting not only the importance of modelling structural flexibility in planar lightsails, but also the possibility of mechanical deformations benefitting lightsail dynamics. Further lowering the spin frequency to 60 Hz also causes the flexible lightsail to veer away from the beam. Denoted times depict either the end of the simulation (2 s) or the time at which the corresponding lightsail reaches $r = 0.2$ m.

Supplementary Note 16: Acceleration performance of flexible metagrating-based lightsail

To calculate the exact acceleration distance to 0.2*c* for the lightsail design shown in Fig. 6, time-domain simulations for the entire acceleration phase would need to be run, which due to reasons of limited computational resources and numerical inaccuracies is currently intractable. At the end of the simulated duration of 5 s, the flat flexible spinning silicon nitride lightsail is accelerated to a velocity of ~1.86 km/s, travelling a distance of ~4.65 km.

Extrapolation to the final velocity of 0.2*c* yields an acceleration distance of ~6.6 Tm for the assumed laser peak intensity of 1 GW/m^2—considerably exceeding the Starshot target, and falling far short in terms of performance compared to other reports. While presenting higher-performing lightsail designs would have been desirable, doing so would have been out of the scope of present research efforts. Instead, this work targeted disclosing methods and techniques to achieve dynamic stability in *flexible* lightsail membranes, which has not yet been shown in the literature.

Motivated by parallel experimental efforts, our designs were restricted to the silicon nitride material system, and to geometries within reach of laboratory fabrication equipment. This decision precluded the consideration of higher-index materials such as MoS_2, which show great promise to dramatically increase the reflectance of lightsails[38] and could likely also reduce the areal mass density required to achieve a self-stabilizing metagrating. Furthermore, we assumed and calculated absorption and emissivity for silicon nitride based upon literature or experimental data. Higher emissivity could eventually be achieved through future photonic engineering[38], or absorption could be reduced through future improvements in fabrication, e.g., during deposition or via thermal annealing. Consequently, this restricted our design to a laser intensity ~10x lower than targeted for Starshot to avoid overheating, required the membrane to be thicker than desired to achieve beam-riding stability, and resulted in relatively low effective reflectance of ~10%. Nevertheless, the simulated design represents a lightsail, which can be fabricated and tested in the lab, and possibly scaled up.

Finally, we note that due to the expected Doppler shift at higher velocities, the optical behavior of our metagratings would differ at later stages of acceleration, to the point where they may not necessarily retain their beam-riding characteristics. Modelling full acceleration dynamics will require further improvements to the simulator code for more robust and efficient calculations, in addition to optical and mechanical characterization of materials over wider wavelength and operating temperature ranges, which is beyond the scope of this manuscript. Nonetheless, we believe that our code and the insights drawn from our results will provide the basis for such improvement and optimization to achieve self-stabilizing, flat flexible lightsails made of silicon nitride for relativistic velocities.

Supplementary References

1. Petersen, K. E. Silicon as a mechanical material. *Proceedings of the IEEE* **70**, 420–457 (1982).
2. Hopcroft, M. A., Nix, W. D. & Kenny, T. W. What is the Young's Modulus of Silicon? *Journal of microelectromechanical systems* **19**, 229–238 (2010).
3. Pearson, G. L., Read Jr, W. T. & Feldmann, W. L. Deformation and fracture of small silicon crystals. *Acta metallurgica* **5**, 181–191 (1957).
4. Kim, J., Cho, D. D. & Muller, R. S. Why is (111) silicon a better mechanical material for MEMS? in *Transducers' 01 Eurosensors XV* 662–665 (Springer, 2001).
5. Hu, S. M. Critical stress in silicon brittle fracture, and effect of ion implantation and other surface treatments. *Journal of Applied Physics* **53**, 3576–3580 (1982).
6. Sharpe, W. N., Yuan, B., Vaidyanathan, R. & Edwards, R. L. Measurements of Young's modulus, Poisson's ratio, and tensile strength of polysilicon. in *Proceedings IEEE the tenth annual international workshop on micro electro mechanical systems. An investigation of micro structures, sensors, actuators, machines and robots* 424–429 (IEEE, 1997).
7. Miyoshi, K. Structures and mechanical properties of natural and synthetic diamonds. (1998).
8. Field, J. E. *Properties of Natural and Synthetic Diamond*. (Academic Press, 1992).
9. Olson, D. S. *et al.* Tensile strength of synthetic chemical-vapor-deposited diamond. *Journal of applied physics* **78**, 5177–5179 (1995).
10. Mohr, M. *et al.* Young's modulus, fracture strength, and Poisson's ratio of nanocrystalline diamond films. *Journal of Applied Physics* **116**, 124308 (2014).
11. Corning® HPFS® 7979, 7980, 8655 Fused Silica. https://www.corning.com/media/worldwide/csm/documents/HPFS_Product_Brochure_All_Grades_2015_07_21.pdf.
12. MATERIALS DATA Quartz Crystal (SiO2) - Crystran. https://www.crystran.co.uk/userfiles/files/quartz-crystal-sio2-data-sheet.pdf.
13. Chao, H. L. & Parker, T. E. *Tensile Fracture Strength of ST Cut Quartz*. (1983).
14. Corning® Gorilla® Glass Victus™. https://www.corning.com/microsites/csm/gorillaglass/PI_Sheets/2020/Corning%20Gorilla%20Glass%20Victus_PI%20Sheet.pdf.
15. Gardon, R. Strong glass. *Journal of Non-Crystalline Solids* **73**, 233–246 (1985).
16. Proctor, B. A., Whitney, I. & Johnson, J. W. The strength of fused silica. *Proceedings of the Royal Society of London. Series A. Mathematical and Physical Sciences* **297**, 534–557 (1967).
17. Edwards, R. L., Coles, G. & Sharpe, W. N. Comparison of tensile and bulge tests for thin-film silicon nitride. *Experimental Mechanics* **44**, 49–54 (2004).
18. Kaushik, A., Kahn, H. & Heuer, A. H. Wafer-level mechanical characterization of silicon nitride MEMS. *J. Microelectromech. Syst.* **14**, 359–367 (2005).
19. Wilson, D. J., Regal, C. A., Papp, S. B. & Kimble, H. J. Cavity Optomechanics with Stoichiometric SiN Films. *Phys. Rev. Lett.* **103**, 207204 (2009).
20. Ftouni, H. *et al.* Thermal conductivity of silicon nitride membranes is not sensitive to stress. *Phys. Rev. B* **92**, 125439 (2015).
21. Bertolazzi, S., Brivio, J. & Kis, A. Stretching and breaking of ultrathin MoS2. *ACS nano* **5**, 9703–9709 (2011).
22. Gu, X., Li, B. & Yang, R. Layer thickness-dependent phonon properties and thermal conductivity of MoS2. *Journal of Applied Physics* **119**, 085106 (2016).

23. Gan, C. K. & Liu, Y. Y. F. Direct calculation of the linear thermal expansion coefficients of MoS 2 via symmetry-preserving deformations. *Physical Review B* **94**, 134303 (2016).
24. Iguiñiz, N., Frisenda, R., Bratschitsch, R. & Castellanos-Gomez, A. Revisiting the Buckling Metrology Method to Determine the Young's Modulus of 2D Materials. *Adv. Mater.* **31**, 1807150 (2019).
25. DuPont Kapton. https://www.dupont.com/content/dam/dupont/amer/us/en/products/ei-transformation/documents/EI-10142_Kapton-Summary-of-Properties.pdf.
26. Friedman, L. Starsailing, solar sails and interstellar travel. *Wiley Science Editions* (1988).
27. McInnes, C. R. *Solar Sailing: Technology, Dynamics and Mission Applications*. (Springer Science & Business Media, 2004).
28. Ma, D., Murray, J. & Munday, J. N. Controllable propulsion by light: steering a solar sail via tunable radiation pressure. *Advanced Optical Materials* **5**, 1600668 (2017).
29. Goubau, G. & Schwering, F. On the guided propagation of electromagnetic wave beams. *IRE Trans. Antennas Propag.* **9**, 248–256 (1961).
30. Brown, W. C. & Eves, E. E. Beamed microwave power transmission and its application to space. *IEEE Trans. Microwave Theory Techn.* **40**, 1239–1250 (1992).
31. Hansen, R. C., McSpadden, J. & Benford, J. N. A universal power transfer curve. *IEEE Microw. Wireless Compon. Lett.* **15**, 369–371 (2005).
32. Parkin, K. L. G. The Breakthrough Starshot system model. *Acta Astronautica* **152**, 370–384 (2018).
33. Manchester, Z. & Loeb, A. Stability of a Light Sail Riding on a Laser Beam. *ApJ* **837**, L20 (2017).
34. Siegel, J. *et al.* Self-Stabilizing Laser Sails Based on Optical Metasurfaces. *ACS Photonics* **6**, 2032–2040 (2019).
35. Hettel, W. *et al.* Beam propagation simulation of phased laser arrays with atmospheric perturbations. *Applied optics* **60**, 5117–5123 (2021).
36. Ilic, O. & Atwater, H. A. Self-stabilizing photonic levitation and propulsion of nanostructured macroscopic objects. *Nat. Photonics* **13**, 289–295 (2019).
37. Ilic, O., Went, C. M. & Atwater, H. A. Nanophotonic Heterostructures for Efficient Propulsion and Radiative Cooling of Relativistic Light Sails. *Nano Lett.* **18**, 5583–5589 (2018).
38. Brewer, J. *et al.* Multiscale Photonic Emissivity Engineering for Relativistic Lightsail Thermal Regulation. *Nano Lett.* **22**, 594–601 (2022).
39. Salary, M. M. & Mosallaei, H. Photonic Metasurfaces as Relativistic Light Sails for Doppler-Broadened Stable Beam-Riding and Radiative Cooling. *Laser & Photonics Reviews* 1900311 (2020) doi:10.1002/lpor.201900311.
40. Santi, G. *et al.* Multilayers for directed energy accelerated lightsails. *Commun Mater* **3**, 16 (2022).
41. Holdman, G. R. *et al.* Thermal Runaway of Silicon-Based Laser Sails. *Advanced Optical Materials* 2102835 (2022) doi:10.1002/adom.202102835.
42. Jaffe, G. R. *et al.* The Effect of Dust and Hotspots on the Thermal Stability of Laser Sails. Preprint at http://arxiv.org/abs/2303.14165 (2023).
43. Rogne, H., Timans, P. J. & Ahmed, H. Infrared absorption in silicon at elevated temperatures. *Applied Physics Letters* **69**, 2190–2192 (1996).
