# Supplementary material for: Dynamically stable radiation pressure propulsion of flexible lightsails for interstellar exploration
Source: Nat Commun. 2024 May 17;15:4203. doi: 10.1038/s41467-024-47476-1 (PMC11101440; doi:10.1038/s41467-024-47476-1)
Supplement: Supplementary file 3 — Description of Additional Supplementary Files [file 41467_2024_47476_MOESM3_ESM.pdf]

Description of Additional Supplementary Files

Four supplementary videos have been prepared to accompany the manuscript, and are available separately from the publisher. They are currently also available on YouTube, linked below.

Supplementary Movie 1

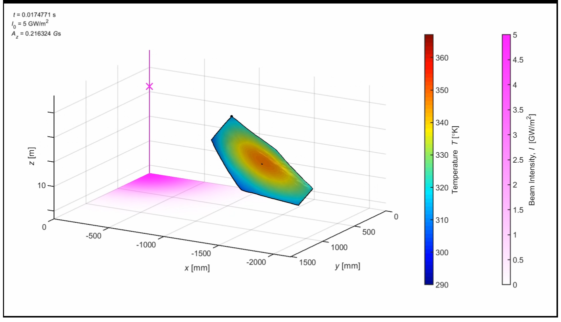

Duration: 1m 44s Resolution: 1920 x 1080 p60

Animation depicts simulations of flat and curved flexible lightsails accelerating in a Gaussian beam, with various initial spin frequencies. These are the simulations shown in Fig. 3 in the manuscript.

Supplementary Movie 2

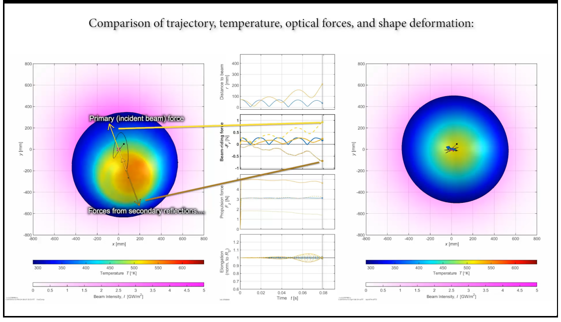

Duration: 2m 0s Resolution: 1920 x 1080 p60

Animation depicts effects of multiple light reflections acting upon curved specular lightsails. Includes the simulations shown in Fig. 4 in the manuscript, as well as several other examples.

Supplementary Movie 3

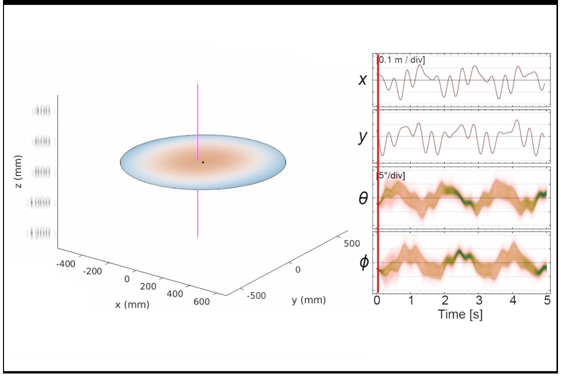

Duration: 1m 0s Resolution: 1200 x 800 p30

Simulation of self-stabilizing flat lightsail based on optical metagratings, accelerating with an initial translational and rotational offset. This simulation is the subject of Fig. 6 in the manuscript.

Supplementary Movie 4

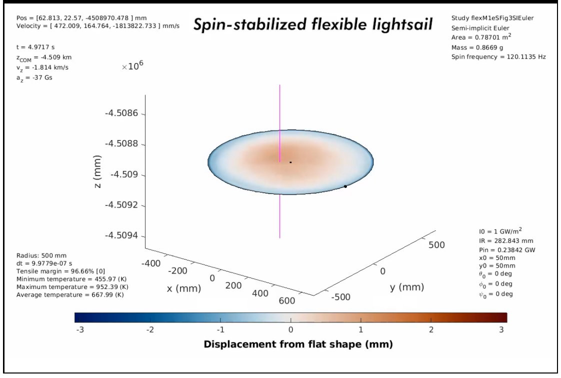

Duration: 45s Resolution: 1200 x 800 p30

Simulation of self-stabilizing flat lightsail based on optical metagratings, accelerating with an initial translational offset only. This simulation is the subject of Supplementary Fig. 10.
